# An AI-Assisted Tool to Predict Continuous Glucose Monitor Adherence in Children With Type 1 Diabetes in Oman: Protocol for a Multiphase Mixed Methods Translational Study

**DOI:** 10.2196/99626

**Published:** 2026-07-13

**Authors:** Thamra Al Ghafri, Saud Al Harthi, Asma Bait Ishaq, Huwaida Al Harthi, Maryam Al Muaini, Rahma Al-Ghadani, Ahmed Aljufaili, Mohamed Al Harb, Abdullah Al Saadi, Abdelhamid Abdessalem, Noushath Shaffi, Mohamed Ahmed, Said Jaboob, Nadia Al Maqbali, Moza Al-Shehhi, Talib Al Kalbani, Amal Al Shukaili, Jannat Al Harthi

**Affiliations:** 1 Directorate General of Health Services Muscat Oman; 2 Sultan Qaboos University Muscat, Muscat Oman; 3 Ministry of Health Directorate General of Khaula Hospital Muscat Oman; 4 Ministry of Health Ministry of Health Batina Oman; 5 Ministry of Health Ministry of Health Sharqiyah Oman; 6 Ministry of Health Ministry of Health AlWosta Oman; 7 Ministry of Health Ministry of Health Dhofar Oman; 8 Ministry of Health Ministry of Health Aldhahira Oman; 9 Ministry of Health Ministry of Health Musandam Oman; 10 Ministry of Health Ministry of Health Buraimi Oman; 11 Ministry of Health Ministry of Health Aldakhilyah Oman; 12 National University of Science and Technology Sohar Oman

**Keywords:** adherence, adolescent, artificial intelligence, behavioral prediction, continuous glucose monitoring, machine learning, pediatric, type 1 diabetes.

## Abstract

**Background:**

Type 1 diabetes mellitus (T1DM) in children requires sustained self-management to achieve glycemic targets. Continuous glucose monitoring (CGM) has transformed pediatric diabetes care; yet, adherence to device wear remains inconsistent. In May 2024, Oman launched a national initiative distributing CGMs to children with T1DM across all governorates, creating a real-world opportunity to study adherence determinants and to develop a locally validated AI-assisted predictive tool.

**Objective:**

This multiphase translational research project aims to (1) characterize the population of Omani children with T1DM; (2) identify demographic, psychosocial, dietary, and physical activity correlates of optimal CGM use; (3) develop, train, and validate an AI-assisted behavioral predictive tool “OMNIdiasense” to forecast CGM adherence prior to device dispensing; and (4) pilot test the OMNIdiasense tool.

**Methods:**

Three sequential, interlinked substudies will be conducted. Substudy 1 is a retrospective cohort analysis of routinely collected Al Shifa data for all children who received CGMs between July 2024 and February 2025, with glycemic, anthropometric, and laboratory outcomes compared at baseline and at ≥3 months. Outputs on adherence prevalence, and clinical predictors become the structured input layer for the proposed AI model. Substudy 2 is a cross-sectional, mixed methods study using face-to-face structured interviews with a randomly selected sample of children aged 10-18 years, classified as “CGM optimizers” (≥6 days/week) or “CGM subusers” (<6 days/week or discontinued); responses across validated behavioral, stress, and dietary instruments are compared. Outputs are the psychosocial and behavioral feature set, qualitative themes, and effect sizes that drive feature selection for the AI model. Substudy 3 develops the AI tool (OMNIdiasense), comprising (1) a quasi-experimental single-arm pilot among 100 existing CGM subusers and (2) a parallel pilot randomized controlled trial (n=50; 25 intervention, 25 control) among newly diagnosed children, with assessments at baseline, 3, 6, and 12 months. The primary outcome is between-group difference in CGM adherence; secondary outcomes include hemoglobin A_1c_, anthropometric, cardiovascular, and laboratory measures, and barriers to use. Reporting will follow SPIRIT (Standard Protocol Items: Recommendations for Interventional Trials) 2025 (interventional component), STROBE (Strengthening the Reporting of Observational Studies in Epidemiology; observational component), the CONSORT (Consolidated Standards of Reporting Trials) 2025 extension for pilot and feasibility trials, COREQ (Consolidated Criteria for Reporting Qualitative Research; qualitative component), and TRIPOD+AI (Transparent Reporting of a Multivariable Prediction Model for Individual Prognosis or Diagnosis + Artificial Intelligence; predictive model).

**Results:**

The proposed AI tool is intended as a decision-support adjunct and not a gatekeeping mechanism for CGM access.

**Conclusions:**

To our knowledge, OMNIdiasense is the first AI tool in the Gulf Cooperation Council region to predict pediatric CGM adherence. By targeting behaviorally vulnerable patients before sensor distribution, OMNIdiasense is expected to support clinical benefit and reduce financial waste.

**Trial Registration:**

ISRCTN Registry ISRCTN15827616; https://doi.org/10.1186/ISRCTN15827616

**International Registered Report Identifier (IRRID):**

DERR1-10.2196/99626

## Introduction

### Background and Significance

Type 1 diabetes mellitus (T1DM) one of the most common chronic conditions of childhood, characterized by autoimmune destruction of pancreatic β-cells and a lifelong dependence on exogenous insulin. Despite the documented rise in incidence, epidemiological data on T1DM remain limited in Oman. A single-center study at Sultan Qaboos University Hospital described 144 Omani children with T1DM, of whom 31% presented in diabetic ketoacidosis [1]. A 2-decade review of diabetes epidemiology in Oman documented an increasing prevalence of pediatric T1DM [2]. The only national incidence study, conducted by Soliman and colleagues [3] over 1993-1994, reported incidence rates of 2.45 and 2.62 per 100,000 person-years, with a peak in the 10-14 years age group and seasonal clustering in cooler months. These figures were lower than those reported in Saudi Arabia (27.5 per 100,000 person-years) and Kuwait (15.4 per 100,000 person-years) [4,5]. The Type 1 Diabetes Index estimates that T1DM in Oman is currently growing at approximately 10.2% per year, outpacing the 7.0% growth of type 2 diabetes [6].

In response to this growing burden, His Majesty Sultan Haitham bin Tarik issued a Royal Directive in February 2024 allocating an annual budget for the Ministry of Health (MoH) to provide continuous glucose monitoring (CGM) devices to Omani children with T1DM [7]. The Ministry began nationwide distribution in May 2024 after extensive capacity-building of diabetes-clinic teams in 30 facilities, with approximately 1500 children receiving CGMs by February 2025. CGM technology has evolved markedly since the United States Food and Drug Administration’s first approval in 1999, with modern systems offering real-time interstitial glucose readings, predictive alerts, and integration with insulin delivery [8,9]. However, the well-documented benefits of CGM on hemoglobin A_1c_ (HbA_1c_) reduction, evident in adults with both type 1 and type 2 diabetes, have not been consistently observed in children and adolescents in early meta-analyses [10].

### CGM in Children: Effectiveness, Adherence, and the Adolescent Gap

Pediatric T1DM trajectories vary by age at onset, genetics, and environment. Younger children may experience more aggressive forms of disease with rapid metabolic deregulation [11], and education for both children and caregivers is central to safe self-management as autonomy increases [12]. Long-term complications, including retinopathy, nephropathy, and cardiovascular disease, can begin during youth, and early-onset cohorts demonstrate distinct trajectories of risk [13].

Recent systematic and randomized evidence increasingly supports CGM in pediatric populations: Teo et al [14] confirmed glycemic improvements with CGM use across age strata; Laffel et al [15] demonstrated superiority of CGM over fingerstick monitoring in adolescents and young adults with elevated HbA_1c_; and Hilliard et al [16] showed parental confidence and satisfaction in young children using CGM. Yet, meta-analytic data continue to highlight cost, accessibility, and educational barriers that erode the population-level effect [17,18]. The 2024-2025 update of the International Society for Pediatric and Adolescent Diabetes (ISPAD) Clinical Practice Consensus Guidelines reaffirms that the effectiveness of real-time CGM in children and adolescents is directly related to the amount of sensor wear and that early initiation following diagnosis is associated with better glycemic outcomes [19].

Despite these advances, registry data show that only about 19% of adolescents in the United States T1D Exchange and 46% in the German/Austrian Diabetes Patienten Verlaufsdokumentation registry meet the previous pediatric HbA_1c_ target of <7.5% [20-22], and the targets have since been further tightened to <7.0% [23,24]. Messer et al [25] introduced the operational distinction between “CGM optimizers” (≥6 days/week) and “CGM subusers” (<6 days/week or discontinuation) and demonstrated that perceived benefit and burden of CGM are the strongest psychosocial predictors of consistent adolescent wear. More recent real-world analyses confirm that adherence is independently associated with HbA_1c_ reduction and lower health care use [26], and 2024-2025 work using the “Adherence Starts with Knowledge-12” questionnaire links pubertal stage, diabetes duration, parental involvement, and BMI SD score with low treatment adherence and worse metabolic control [27].

### AI to Predict and Support CGM Adherence

Machine learning and deep learning models trained on wearable and CGM-derived signals are increasingly used to forecast glucose trajectories, detect lifestyle patterns, and personalize care [28-30]. AI-based decision support systems for T1DM have demonstrated feasibility and potential to refine insulin dosing and behavioral guidance [31,32]. A 2024 systematic review identified 8 domains in which AI is reshaping diabetes care, including health monitoring, predictive modeling, lifestyle and dietary management, clinical decision-making, and patient engagement [33]. Personalized blood glucose prediction models combining bidirectional long short-term memory networks with transformer architectures and meta-learning recently achieved clinically acceptable accuracy in pediatric and adult T1DM datasets [34]. To date, however, no AI tool has been developed and validated in the Gulf Cooperation Council region to predict CGM adherence prior to device dispensing in children, and no such tool has been integrated with motivational interviewing (MI)–based behavior change support.

### Study Rationale

The Omani national CGM rollout is pragmatic but resource-intensive, and suboptimal device use risks attenuating both clinical benefit and cost-effectiveness. A locally developed predictive tool, informed by real-world Omani data and culturally adapted psychosocial constructs, could enable risk stratification before CGM dispensing, allowing targeted behavioral support for predicted subusers while reducing waste among children unlikely to engage. This protocol describes a coordinated 3-substudy program to build, validate, and pilot such a tool “OMNIdiasense” in service of His Majesty’s Royal Directive.

### Objectives

The primary objective of this study is to conduct formative research to inform the design of OMNIdiasense, an AI-assisted assessment tool that predicts CGM adherence among Omani children aged 10-18 years with T1DM, and to develop and pilot-test the tool prior to any consideration of national implementation. The study also aims to optimize CGM use through personalized, targeted behavioral interventions for children predicted to be subusers and to estimate the potential cost reduction associated with the implementation of OMNIdiasense. The secondary objectives are to identify the challenges associated with current CGM use among Omani children with T1DM, optimize health care service delivery processes for CGM provision, map the value stream of CGM prescribing and dispensing, identify financial waste related to CGM use through cost analysis, integrate lean management methods into CGM service delivery, and build the capacity of health care workers in the effective use of pediatric CGM technologies.

## Methods

### Overall Study Design and Setting

**Figure 1:**
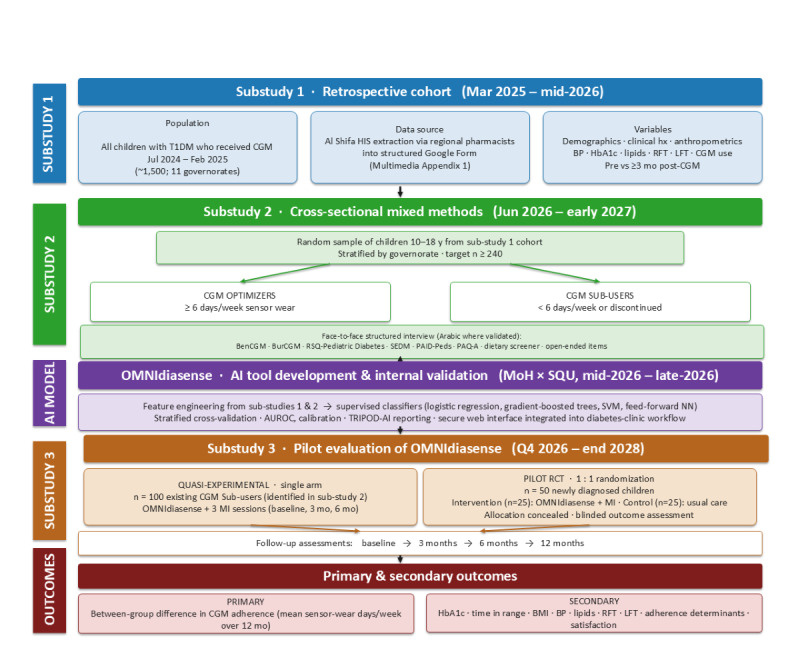
Multiphase study flow of the OMNIdiasense project. AUROC: area under the receiver operating characteristic curve; BenCGM: Benefits of Continuous Glucose Monitoring Scale; BP: blood pressure; BurCGM: Burden of Continuous Glucose Monitoring Scale; CGM: continuous glucose monitoring; HbA1c: hemoglobin A1c; HIS: health information system; LFT: liver function test; MoH: Ministry of Health; NN: neural network; PAID: Problem Areas in Diabetes; PAQ-A: Physical Activity Questionnaire for Adolescents; RFT: renal function test; RSQ: Response to Stress Questionnaire; SEDM: Self-Efficacy for Diabetes Management Scale; SQU: Sultan Qaboos University; SVM: support vector machine; T1DM: type 1 diabetes mellitus; TRIPOD+AI: Transparent Reporting of a Multivariable Prediction Model for Individual Prognosis or Diagnosis + Artificial Intelligence.

This is a multiphase, 2-year (2025-2027) translational research project conducted across all 11 governorates of the Sultanate of Oman. The project comprises 3 interlinked substudies: (1) a retrospective cohort study, (2) a cross-sectional mixed methods study, and (3) a pilot evaluation combining a single-arm quasi-experimental design with a parallel pilot randomized controlled trial (RCT). Outputs from substudies 1 and 2 will provide the data and theoretical constructs required to develop the AI-assisted OMNIdiasense tool tested in substudy 3. Figure 1 presents the overall study schematic, and Table 1 summarizes the expected outcomes of each substudy.

**Table 1:** Description of the substudies by design, outcomes, and how each informs the next.

Substudy and aim	Design	Primary outputs/outcomes	How it informs the next phase
Substudy 1: characterize population	Retrospective cohort (Al Shifa records, n~1500)	Population characterization; pre/post HbA_1c_^a^, anthropometrics, BP^b^, lipids, RFT^c^, LFT^d^; sensor-wear days per week; baseline adherence prevalence	Provides the deidentified structured dataset (clinical + adherence label) used as the supervised-learning input for OMNIdiasense; defines candidate clinical predictors.
Substudy 2: identify correlates	Cross-sectional mixed methods (n=240 face-to-face interviews + qualitative open-ended responses)	Quantitative effect sizes for BenCGM^e^, BurCGM^f^, RSQ^g^, SEDM^h^, PAID-Peds^i^, PAQ-A^j^, and dietary screener on optimizer vs subuser status; qualitative themes on barriers and facilitators	Provides the psychosocial and behavioral feature set, the qualitative themes that inform MI content, and the effect sizes used to prioritize features for the AI model.
Substudy 3: AI tool development (7-step pipeline with validation hierarchy)	Prediction model development on combined substudy 1 + 2 dataset	OMNIdiasense risk score (probability of subuser status), with discrimination, calibration, and fairness metrics	The validated model becomes the assessment tool tested in the 2 evaluation arms below.
Substudy 3: quasi-experimental single-arm pilot	Quasi-experimental, n=100 existing subusers	Within-person change in adherence and HbA_1c_ after OMNIdiasense-guided MI sessions	Feasibility, acceptability, and effect-size estimates for a future definitive trial.
Substudy 3: pilot RCT^k^ with appropriate reporting standards	Parallel 1:1, n=50 newly diagnosed (25 intervention, 25 control)	Between-group difference in CGM^l^ adherence at 12 months; secondary clinical, behavioral, and satisfaction outcomes	Generates the effect-size, recruitment, retention, and fidelity estimates required for a definitive multicenter trial.

^a^HbA_1c_: hemoglobin A_1c_.

^b^BP: blood pressure.

^c^RFT: renal function test.

^d^LFT: liver function test.

^e^BenCGM: Benefits of Continuous Glucose Monitoring Scale.

^f^BurCGM: Burden of Continuous Glucose Monitoring Scale.

^g^RSQ: Response to Stress Questionnaire.

^h^SEDM: Self-Efficacy for Diabetes Management Scale.

^i^PAID-Peds: Problem Areas in Diabetes-Pediatric version.

^j^PAQ-A: Physical Activity Questionnaire for Adolescents.

^k^RCT: randomized controlled trial.

^l^CGM: continuous glucose monitoring.

CGM distribution in Oman commenced in May 2024 following extensive capacity-building of diabetes clinic staff in 30 health facilities across all governorates. By February 2025, approximately 1500 children with T1DM had received CGMs, recorded centrally in the national Al Shifa health information system.

### Coordination and Workforce

A total of 11 regional research managers (RMs), senior pharmacists nominated by their respective directorates general of health services, will coordinate the project. Their responsibilities will include:

- Facilitating data collection in alignment with substudy objectives.
- Supervising the local conduct of the project.
- Liaising with families and clinical teams.
- Coordinating with relevant departments and sections regionally.
- Resolving operational queries.

All RMs will receive standardized training on study assessment tools, ethical considerations, conflict resolution, and data protection prior to data collection.

A trial steering committee, comprising the principal investigators, 2 regional directorate representatives, an independent statistician, and a parent representative, will meet quarterly to oversee progress and protocol fidelity. An independent data monitoring committee (DMC) of 3 external members (a senior clinical practitioner, a biostatistician, and a quality officer) will review safety, recruitment, and data-quality reports every 6 months. The DMC will operate under a charter agreed upon before recruitment begins.

### Substudy 1: Characteristics and Glycemic Outcomes of Omani Children Receiving CGMs

#### Design and Population

Substudy 1 is a retrospective cohort analysis of all Omani children with T1DM who received CGM through the national rollout between July 2024 and February 2025 across the 11 governorates. The minimum analytic exposure window is 3 months of CGM use from baseline.

#### Conceptual Background

T1DM in children is heterogeneous, and trajectories of glycemic control depend on age at onset, family history, comorbidities, and self-management capacity [11-13]. CGM has been shown to improve glycemic control in children when wear is consistent [14-16,19]. Quantifying baseline characteristics and short-term post-CGM outcomes in the Omani population will inform both clinical follow-up pathways and the variable set used to train OMNIdiasense.

#### Expected Outcomes

Primary outcomes of substudy 1 are (1) the population-level prevalence of CGM optimizer status at ≥3 months and (2) the pre-post change in HbA_1c_. Secondary outcomes are pre-post changes in weight, BMI, BMI *z* score, systolic and diastolic blood pressure, lipid panel, renal function test (RFT), and liver function test (LFT); regional variation across governorates; and identification of routinely captured clinical predictors of optimal use that will be carried forward to the AI model in substudy 3.

#### Data Collection

Regional pharmacists, supported by their RMs, will extract the following data from Al Shifa for each eligible child into a structured Google Form (Multimedia Appendix 1):

- Demographic characteristics
- Medical history of T1DM
- Family history of diabetes (type and degree)
- Comorbidities
- Drug and treatment history (pre-CGM and ≥3 months post-CGM)
- Anthropometric measures (pre and post)
- Physiological measures, including blood pressure (pre and post)
- Laboratory investigations: lipid panel, RFT, LFT, HbA_1c_ (pre and post)
- CGM device type, cost, and number of dispensations
- Duration of CGM wear (≥6 days/week vs <6 days/week)

#### Statistical Analysis

Descriptive statistics will be presented as frequencies, percentages, means, and SDs or medians and IQRs, as appropriate. Pre-post comparisons will use paired *t* tests or Wilcoxon signed-rank tests for continuous variables and McNemar tests for categorical variables. Differences across governorates will be evaluated with chi-square tests and ANOVA. Logistic regression will model predictors of optimal CGM use. Analyses will be performed in IBM SPSS Statistics (version 28) and R software (version 4.4; R Foundation for Statistical Computing). Reporting will follow STROBE (Strengthening the Reporting of Observational Studies in Epidemiology) guidelines (Multimedia Appendix 2).

### Substudy 2: Correlates of CGM Adherence: Cross-Sectional Mixed Methods Study

#### Design and Population

Substudy 2 is a cross-sectional mixed methods study using supervised face-to-face interviews with adolescents aged 10-18 years drawn from substudy 1. RMs will randomly sample participants from the Al Shifa–derived cohort. The dependent variable, pattern of CGM use, will be dichotomized as CGM optimizers (≥6 days/week) and CGM subusers (<6 days/week or discontinued), consistent with prior pediatric work [25].

Eligible participants will include children aged 10-18 years with T1DM who received CGM under the national initiative and whose primary caregiver consents. Children will be excluded if they are unable to complete the structured interview owing to severe cognitive or sensory impairment.

#### Conceptual Framework

Despite CGM’s technical maturity, the majority of adolescents with T1DM do not meet glycemic targets [20-24]. Self-management decisions are shaped by perceived benefit and burden of CGM, self-efficacy, coping responses, diabetes distress, dietary patterns, and physical activity [25,27]. Substudy 2 measures these constructs and their open-ended elaborations to inform feature selection for OMNIdiasense.

#### Expected Outcomes

Primary outcomes of substudy 2 are the standardized mean differences and odds ratios for each psychosocial and behavioral instrument (Benefits of Continuous Glucose Monitoring Scale [BenCGM], Burden of Continuous Glucose Monitoring Scale [BurCGM], Response to Stress Questionnaire-Pediatric Diabetes [RSQ-PD] coping factors, Self-Efficacy for Diabetes Management Scale [SEDM], Problem Areas in Diabetes-Pediatric version [PAID-Peds], Physical Activity Questionnaire for Adolescents [PAQ-A], and dietary screener) between optimizers and subusers. Secondary outcomes are the qualitative themes generated from open-ended responses, the convergent mixed methods joint display, and the ranked feature list passed to substudy 3 for model training (Multimedia Appendix 3).

#### Sample Size

Assuming an optimizer prevalence of approximately 50% [25] and aiming to detect medium-effect-size predictors in a multivariable logistic model with up to 8 predictors, a minimum of 240 participants will provide sufficient statistical power (≥10 events per variable). Recruitment will be stratified across the 11 governorates proportional to CGM dispensations.

#### Measurements

The face-to-face interview will include the following components, administered in Arabic where validated translations are available (Multimedia Appendix 4). Questionnaires that require official approval from the original authors or involve payment or restricted use will be obtained accordingly. Components of the questionnaire include the following:

- Demographics and diabetes management: age, gender, region, current insulin modality (multiple daily injections vs insulin pump), past or current CGM use, duration of CGM use; diabetes duration imported from substudy 1.
- BenCGM and BurCGM: 8-item Likert scales each, validated in adolescents aged 12-19 years [25,35]. Cronbach α will be reestimated in a pilot phase.
- RSQ-PD: 57-item adolescent-specific measure yielding 5 proportional coping/stress factors [36-38].
- SEDM: 10-item single-factor scale validated in T1DM adolescents 13-17 years [39,40].
- PAID-Peds: 20-item measure of diabetes distress in youth [41].
- PAQ-A, Arabic version [42].
- Short dietary history screener [43].
- Open-ended items: compliance with CGM, school and educational performance, barriers and facilitators, side effects, and recommendations.

#### Data Analysis

Quantitative analyses will compare optimizers and subusers using chi-square tests, *t* tests, and Mann-Whitney *U* tests, with multivariable logistic regression to identify independent psychosocial and behavioral predictors of optimal CGM use. Qualitative responses to open-ended items will be audio-recorded with consent, transcribed verbatim, and analyzed in NVivo (Lumivero) by 2 independent coders (1 bilingual Arabic-English nurse researcher and 1 public health researcher) using a hybrid inductive-deductive approach; discrepancies will be resolved by a third senior coder, and a 20% subsample will be member-checked for accuracy. Field notes will be written within 24 hours of each interview. Findings will be triangulated using a convergent mixed methods design with joint displays. Reporting will follow STROBE (Multimedia Appendix 2) for the quantitative component and COREQ (Consolidated Criteria for Reporting Qualitative Research; Multimedia Appendix 5) for the qualitative component.

### Substudy 3: Pilot Evaluation of OMNIdiasense

#### Design

Substudy 3 evaluates the AI-assisted assessment tool OMNIdiasense through 2 parallel components, both fed by an embedded AI model-development workstream described below.

1. Quasi-experimental single-arm pilot: 100 existing CGM subusers identified in substudy 2 receive OMNIdiasense assessment prior to CGM reintroduction, followed by MI-guided behavioral support delivered by a trained diabetic nurse within the routine pediatrics endocrine clinics.
2. Pilot RCT: 50 newly recruited children with T1DM, randomized 1:1 to (1) intervention, OMNIdiasense assessment plus MI-guided support before CGM initiation or (2) usual care without exposure to the tool. Both arms receive CGM and standard clinical follow-up.

All 150 participants are followed for 12 months, with assessments at baseline, 3, 6, and 12 months. The flow of participants is presented in Figure 1.

#### AI Model Development (OMNIdiasense)

The AI tool will be codeveloped by the MoH digital-health team and computer scientists from Sultan Qaboos University. The development pipeline comprises 7 steps, expanded below to address missing data, class imbalance, overfitting, feature selection, calibration, fairness, and external validation.

- Data preparation: integration and cleaning of deidentified data from substudies 1 and 2 (demographic, clinical, laboratory, anthropometric, psychosocial, and behavioral).
- Feature engineering: selection of candidate predictors informed by substudy 2 effect sizes and clinical face validity.
- Model selection and training: comparison of supervised classifiers (logistic regression, gradient-boosted trees, support vector machines, and a small feed-forward neural network) using stratified cross-validation; evaluation by area under the receiver operating characteristic curve, sensitivity, specificity, calibration, and net reclassification improvement.
- Behavioral pattern recognition: unsupervised clustering of lifestyle and dietary data to derive interpretable phenotypes used for personalized recommendations.
- Initial testing: internal validation on a held-out 20% partition.
- Optimization: iterative refinement on the basis of clinician and family feedback.
- Deployment: secure web-based interface integrated into the diabetes clinic workflow with role-based access and audit logging.

#### Handling of Missing Data, Class Imbalance, Overfitting, and Feature Selection

Missingness will be quantified by variable and pattern. Variables with >40% missingness will be excluded unless they carry strong prior clinical value, in which case they will be retained with an explicit missing indicator. Item-level missing data on validated scales will be imputed using each scale’s published rules; remaining missingness will be addressed by multiple imputation by chained equations (m=20) under a missing-at-random assumption, with a tipping-point sensitivity analysis to test robustness against a missing-not-at-random scenario.

Class imbalance between optimizers and subusers will be addressed at 2 levels: stratified sampling during cross-validation to preserve outcome proportions in every fold, and class-weighted loss functions (inverse-frequency weights) for the gradient-boosted, SVM, and neural-network classifiers. Synthetic minority oversampling will be evaluated as a sensitivity analysis only and not as the primary approach, to avoid optimistic generalization.

Overfitting will be controlled through nested cross-validation (outer 5-fold for performance estimation, inner 5-fold for hyperparameter tuning), L1/L2 regularization for linear models, early stopping and shrinkage for boosted trees, and dropout and weight decay for the neural network. The full train-test partition (80/20) will be temporally separated where possible (children enrolled later become the held-out set) to give a more realistic estimate of prospective performance.

Feature selection will use a stability-selection wrapper around L1-penalised logistic regression with 100 subsampled fits; features selected in ≥60% of subsamples will be retained, in addition to a clinically prespecified core set (age, sex, diabetes duration, baseline HbA_1c_, insulin modality, governorate, BenCGM, BurCGM, SEDM, and PAID-Peds). The final model will be sparse and interpretable; Shapley additive explanations (SHAP) values will be reported to support clinical interpretation of individual predictions.

#### Calibration

Discrimination alone is insufficient when a model is used to triage clinical support. Calibration will be assessed using calibration plots, the Brier score, calibration-in-the-large, and calibration slope. If the raw model is miscalibrated, isotonic regression or Platt scaling will be applied to the inner cross-validation folds and reevaluated on the outer fold. Decision-curve analysis across plausible threshold probabilities will accompany the calibration report.

#### Fairness Across Sex, Age, and Governorate

Fairness will be evaluated as a primary model-quality dimension, not as an afterthought. For each protected attribute (sex, age band 10-13 years vs 14-18 years, and governorate), we will report subgroup-specific area under the curve, sensitivity, specificity, calibration slope, and false-negative rate (the most consequential error, since a false negative could deny behavioral support to a child who needs it). Equality of opportunity and predictive parity will be reported. If a clinically meaningful disparity is detected (absolute difference in false-negative rate >10 percentage points), we will apply group-specific threshold adjustment and reevaluate before deployment.

#### External Validation

Internal validation alone is insufficient before clinical use. The validation plan therefore includes three layers: (1) internal validation on the held-out 20% partition, (2) temporal validation on a prospectively collected cohort enrolled at least 3 months after model lock, and (3) a preplanned external multisite replication in 2 Gulf neighboring sites (institutions to be confirmed at the start of the pilot phase) using the same data-collection forms and the same outcome definitions. Until external validation is satisfactory, OMNIdiasense outputs will be presented to clinicians as decision support only and will not influence CGM allocation (see the Ethical Considerations section).

#### Reporting Standard

Reporting of the AI development will follow the TRIPOD+AI (Transparent Reporting of a Multivariable Prediction Model for Individual Prognosis or Diagnosis + Artificial Intelligence) guideline [44,45], which provides a 27-item checklist for prediction-model studies that use traditional regression or AI/machine learning methods. The TRIPOD+AI checklist will be uploaded with the results paper.

#### Personalization and Feedback Mechanism

OMNIdiasense will generate a per-patient adherence-risk score with personalized, MI-aligned recommendations covering meal timing, physical activity, stress management, and parental engagement. The system will be piloted in Muscat for 6 months prior to wider testing.

#### Sample Size

The pilot RCT sample size was based on the prevalence of optimal CGM use of approximately 50% [25] and a moderate effect (odds ratio 0.53) reported for adolescent CGM use. Using G*Power 3.1 (Heinrich Heine University Düsseldorf), at least 25 participants per arm are required to achieve 80% power at α=.05. The single-arm component (n=100) is powered for paired-sample within-person change in adherence and HbA_1c_.

#### Inclusion and Exclusion Criteria

Quasi-experimental component: existing children with T1DM aged 10-18 years classified as CGM subusers in substudy 2; primary caregiver consents and child assents.

RCT component: newly diagnosed children with T1DM, aged 10-18 years, eligible for CGM under the national program, who have not yet received CGM. Exclusion: severe psychiatric or cognitive comorbidity precluding informed assent or interview participation.

#### Recruitment

Eligible families will receive a formal invitation letter, information sheet, and consent/assent forms (Multimedia Appendix 6) from RMs, who will explain study objectives and logistics in Arabic and English.

#### Training

A 3-day bespoke training program will be delivered to RMs, diabetes nurses, and administrative staff, covering the OMNIdiasense interface, MI principles, outcome measurement procedures, and ethical considerations.

#### Intervention

Participants in the quasi-experimental arm and the RCT intervention arm will receive 3 MI-guided consultations delivered by trained diabetes nurses at baseline, 3 months, and 6 months (Multimedia Appendix 7). MI sessions will (1) establish rapport, (2) explore pros and cons of CGM use, (3) provide tailored feedback on the OMNIdiasense risk score, (4) elicit imagery of life with and without CGM, (5) review options and develop an adherence plan, and (6) reinforce self-efficacy.

Permitted concomitant care includes routine pediatric diabetes follow-up, school health support, and standard insulin therapy. Discontinuation of OMNIdiasense recommendations will be considered if the family withdraws consent, if a clinical adverse event makes continued sensor wear unsafe, or if the treating clinician determines that a recommendation conflicts with safety. Adherence to the MI fidelity manual will be assessed by audio-recorded session sampling (approximately 10% of sessions per interventionist per quarter), scored using a brief MI fidelity rubric (Multimedia Appendix 8).

#### Outcomes

The primary outcome is the between-group difference in CGM adherence, defined as mean days per week of sensor wear over the 12-month follow-up. The CGM adherence outcome will be measured from pharmacy dispensing records, self-report, and clinical records. Differences between device types will be recorded, and significant findings across different devices will be analyzed.

Key secondary outcomes are change in HbA_1c_, time in range, anthropometric measures (weight and BMI), blood pressure, lipid panel, RFT, LFT, drug/treatment changes, OMNIdiasense risk score change, barriers and facilitators of CGM use, and patient satisfaction (Multimedia Appendix 9). Table 2 shows the full schedule of assessments.

**Table 2:** Schedule of assessments in substudy 3.

Variable		Tool/source	Baseline	3 months	6 months	12 months
**Primary outcome**						
	CGM^a^ adherence (optimizer vs subuser)	OMNIdiasense score	✓	✓	✓	✓
	Sensor-wear days/week	CGM device records	✓	✓	✓	✓
	MI^b^-guided behavior change consultation	Session log	✓		✓	✓
**Secondary outcomes**						
	Demographic characteristics	Study questionnaire	✓			
	Medical history of T1DM^c^	Study questionnaire	✓			
	Family history of diabetes	Study questionnaire	✓			
	Comorbidities	Study questionnaire	✓			
	Drug/treatment history	Health records	✓		✓	✓
	Anthropometrics (weight, BMI)	Health records	✓		✓	✓
	Blood pressure	Health records	✓		✓	✓
	Cholesterol, RFT^d^, LFT^e^	Health records	✓		✓	✓
	HbA_1c_^f^	Health records	✓		✓	✓
	Barriers and facilitators	Open-ended interview	✓		✓	✓
	Patient satisfaction	Satisfaction questionnaire			✓	✓
	Staff satisfaction (exit survey)	Satisfaction questionnaire				✓

^a^CGM: continuous glucose monitoring.

^b^MI: motivational interviewing.

^c^T1DM: type 1 diabetes mellitus.

^d^RFT: renal function test.

^e^LFT: liver function test.

^f^HbA_1c_: hemoglobin A_1c_.

#### Randomization and Blinding

In the RCT component, computer-generated 1:1 simple randomization will be performed centrally by an independent statistician. Allocation concealment will use sequentially numbered, opaque, sealed envelopes opened only after enrollment confirmation. Outcome assessors and the data analyst will be blinded to allocation; participants and intervention deliverers will not be blinded owing to the nature of the intervention.

#### Data Analysis

Analyses will follow the intention-to-treat principle. Descriptive statistics will summarize baseline characteristics. Within-person change in the quasi-experimental arm will be analyzed with paired *t* tests, Wilcoxon signed-rank tests, or generalized estimating equations as appropriate. Between-group differences in the RCT will use *t* tests, chi-square tests, and mixed effects models with random intercepts for repeated measures. Multivariable linear and logistic regressions will explore predictors of adherence change. Missing data will be handled by multiple imputation under a missing-at-random assumption with sensitivity analyses. Prespecified subgroup analyses will examine age band (10-13 vs 14-18 years), sex, governorate (Muscat vs others), and insulin modality (multiple daily injections vs pump); any post hoc subgroup analyses will be flagged as exploratory. The pilot RCT report will follow the CONSORT (Consolidated Standards of Reporting Trials) 2025 extension for pilot and feasibility trials, and the interventional protocol adheres to SPIRIT (Standard Protocol Items: Recommendations for Interventional Trials)-AI 2025 (Multimedia Appendix 10).

#### Safety, Harms, and Auditing

All adverse device effects (eg, persistent skin reactions and false hypoglycemia alarms with clinical sequelae) and serious adverse events will be solicited at every study visit and through a dedicated 7-day reporting channel. Events will be graded using the United States Food and Drug Administration Medical Device Reporting framework, reported to the DMC, and forwarded to the regional patient safety and quality departments. Independent audits of consent, source data, and protocol adherence will be performed at 6 and 18 months by the National Center of Studies and Research.

#### Protocol Amendments and Stopping Rules

Substantive protocol amendments will be submitted to the MoH ethics committee for evaluation and approval. Prespecified stopping rules include (1) a device-related serious adverse event rate of 10% or greater, (2) a DMC recommendation on safety or futility grounds, or (3) interruption of national CGM supply.

#### How the OMNIdiasense Score Will Be Used

OMNIdiasense generates a per-patient probability of becoming a CGM subuser. Throughout the studies described in this protocol, this score is used only as a decision-support adjunct to trigger MI-guided behavioral support and to personalize the content of that support. The score will not be used to deny, delay, or withdraw CGM access. CGM allocation will continue to follow the existing national clinical pathway determined by the treating pediatric diabetes team; the score is recorded in the research database, not in the patient’s clinical record, during the pilot phase.

#### Communication of Risk Predictions to Families

Risk predictions will be communicated using verbal categorical language (“higher,” “moderate,” and “lower”) and not as raw probabilities. The communication template, developed with input from the parent advisory group, includes (1) an explanation that the score is a research-stage estimate, not a diagnosis, (2) an explanation that the score does not affect CGM access, (3) the actionable behavioral recommendations linked to that score, and (4) the family’s right to decline the recommendations without consequence for routine care. Counseling is delivered by the trained diabetes nurse, with the treating clinician available for clinical questions.

#### Data Privacy and Information Governance

Identifiable data are stored only within the MoH Al Shifa environment behind the national firewall. The analytic dataset shared with the AI team is pseudonymized: the link key is held by the principal investigator in a separate encrypted store. Access is role-based and audit-logged. Model training and inference occur in a MoH-controlled environment; no patient data leaves the national network for model training or inference. Data sharing for external validation in Gulf neighboring sites will use federated analysis (aggregate parameter exchange) rather than transfer of individual-level records, where technically feasible, and otherwise a data transfer agreement compliant with Omani Personal Data Protection Law (Royal Decree 6/2022) and the host country’s equivalent will govern any transfer [46].

#### Bias Monitoring During the Pilot

Subgroup performance (sex, age band, governorate, and insulin modality) will be reviewed by the DMC at 6-month intervals during the pilot. If a meaningful subgroup disparity is detected, deployment of model outputs for that subgroup will be paused while the model is recalibrated, and the DMC will advise on whether to resume. A bias monitoring log will be kept and included in the results paper.

#### Algorithmovigilance and Postdeployment Monitoring

Beyond the pilot, any future implementation will be conditional on (1) satisfactory external validation, (2) ethics reapproval, and (3) an algorithmovigilance plan covering version control, drift monitoring (population shift in predictors and outcomes), threshold review, complaint handling, and a clearly documented decommissioning pathway. The decommissioning pathway includes the conditions under which the tool would be withdrawn (eg, calibration drift, disparity, and supersession by a better tool) and the steps to remove it without disrupting routine care.

### Patient and Public Involvement

The study was designed in alignment with a national CGM rollout decided at the policy level; consequently, patients and members of the public were not involved in its initial design. However, parental and adolescent input will be incorporated during the OMNIdiasense user-interface refinement phase and in the interpretation and dissemination of findings. A parent advisory group of 5 caregivers will be convened in the first quarter of substudy 2 to review interview content and dissemination products.

### Reporting Guidelines and Dissemination

The protocol adheres to the following reporting guidelines: SPIRIT 2025 (Multimedia Appendix 10) for the interventional component [44,47], CONSORT 2025 extension for pilot and feasibility trials, STROBE for observational components, COREQ for qualitative components, and TRIPOD+AI for the predictive model [46,47]. Completed checklists will be uploaded as multimedia appendices on submission of subsequent results papers.

Participant-facing dissemination will include an Arabic-language plain-language summary handed to enrolled families, clinician dissemination through national pediatric diabetes meetings, and academic dissemination through peer-reviewed journals and conference presentations. The full statistical analysis plan can be made available upon request to the principal investigator and with approvals from all members of the research team. No professional medical writers will be used.

### Dissemination Plan

Academic dissemination will target journals indexed in MEDLINE that cover pediatric endocrinology, digital health, and implementation science. Conference dissemination will include the ISPAD annual meeting, the European Association for the Study of Diabetes (EASD) Technology Symposium, and the Gulf Diabetes Conference. Policy dissemination will target the MoH Directorate General of Planning and Studies and the National Health Research Center, in the form of an executive summary and a costed implementation brief. Public dissemination will use the MoH’s communications channels and the Omani Diabetes Association in Arabic and English. Participants will receive an Arabic-language plain-language summary of the main findings; substudy 2 qualitative quotes will only be used in anonymized form, with reconsent for any direct quotation in dissemination products.

### Ethical Considerations

The Oman Research and Ethical Review and Approve Committee, MoH, granted ethical approval for the overall protocol (MoH/CSR/24/29506; Multimedia Appendix 11). Each substudy additionally undergoes independent ethical review. Parental written informed consent and child written assent (where age-appropriate) will be obtained prior to any study procedure. Withdrawal at any point is permitted without effect on routine care. Personally identifiable data will be stored in encrypted MoH systems with role-based access; analytic datasets will be deidentified, and reidentification keys will be held by the principal investigator only. The trial is registered with International Standard Randomized Controlled Trial Number (ISRCTN; ISRCTN15827616). The protocol adheres to the 2024 Declaration of Helsinki and Good Clinical Practice (ICH-GCP E6 R3). No financial incentives are provided beyond reimbursement for travel where applicable. In the unlikely event that a participant experiences harm directly attributable to study procedures, posttrial care will be provided through routine MoH services at no additional cost to the family; no separate financial compensation scheme is offered.

## Results

The project was funded in March 2025 by the Ministry of Higher Education, Research and Innovation through the Strategic Research Program, supervised by the National Health Research Center (agreement number RIA/SRP/MoH/25/01; Multimedia Appendix 6). Ethical approval was issued in November 2024 (MoH/CSR/24/29506), and the trial was prospectively registered with ISRCTN on April 22, 2025 (ISRCTN15827616) (Multimedia Appendix 12). Data extraction from Al Shifa for substudy 1 began in March 2025; as of April 2026, baseline records for the recruited sample have been compiled, and cleaning and pre-post comparative analyses are underway. Substudy 2 interview tools have been prepiloted in Muscat with 15 adolescents in February 2026, and full recruitment is planned to begin in June 2026. AI model prototyping with synthetic data and feature engineering using substudy 1 outputs is scheduled for the third quarter of 2026; substudy 3 pilot recruitment is planned for the fourth quarter of 2026, with the final 12-month follow-up expected to conclude by the end of 2028. Findings from substudies 1 and 2 are anticipated for publication in mid-2027, the OMNIdiasense development paper, and the pilot evaluation results in late 2027.

## Discussion

### Anticipated Principal Findings

We anticipate 3 principal findings. First, substudy 1 is expected to show that pediatric CGM uptake in Oman is high in the first 6 months of dispensing, but that a clinically important minority becomes subusers by month 3, with measurable variation across governorates. Second, substudy 2 is expected to confirm prior international findings that perceived benefit and burden of CGM, self-efficacy, and diabetes distress are the strongest psychosocial correlates of optimizer status, with additional Omani-specific contributors emerging from the open-ended responses. Third, substudy 3 is expected to show that OMNIdiasense, used as a decision-support adjunct followed by MI-guided behavioral sessions, is feasible to deliver, acceptable to families and clinicians, and produces a clinically meaningful between-group difference in adherence at 12 months that can power a future definitive trial.

These anticipated findings are framed as testable hypotheses, not foregone conclusions, and all statements throughout the abstract, manuscript, and conclusion have been rechecked to ensure that the strength of language matches the strength of inference that the proposed methodology can support.

### Comparison With Prior Work

Existing AI work in T1DM has focused predominantly on glucose forecasting [29,30,34], adult populations [28,31,32], or insurance-based CGM adherence in high-income settings [26]. Our protocol differs in three respects: (1) it targets adherence prior to device dispensing, rather than after nonadherence has emerged; (2) it integrates AI prediction with MI-based behavioral consultations as a single care pathway; and (3) it situates the work within a publicly funded national rollout in a Middle Eastern, predominantly Arabic-speaking pediatric population, addressing a substantial gap in geographic and demographic representation [33].

In contrast to commercial CGM-adherence analytics described in claims-based datasets [26], OMNIdiasense is designed to be transparent (sparse model and SHAP-based explanations), publicly funded, locally owned, and explicitly evaluated for fairness across sex, age, and governorate before any consideration of clinical deployment.

### Strengths and Limitations

Strengths of this protocol include national-scale recruitment across all 11 governorates, prospective alignment with SPIRIT 2025, CONSORT 2025, STROBE, COREQ, and TRIPOD+AI [45,48], the use of multiple validated psychosocial instruments, an explicit fairness and external-validation plan, and a coordinated research-management infrastructure that mirrors the operational structure of the MoH.

Anticipated limitations include reliance on routinely collected data with potential for missingness in substudy 1, possible social-desirability bias in face-to-face interviews in substudy 2, modest sample size in the pilot RCT, single-country generalizability, and the absence of patient and public involvement in initial design. The 12-month follow-up may be too short to detect changes in long-term complications, and the AI tool will require external validation in additional Gulf populations before scale-up. The pilot effect sizes will require confirmation in a definitive multisite trial, and the planned subgroup-fairness audit may itself be underpowered for rare strata.

### Future Directions

If the pilot results are favorable, the next step would be a definitive multisite Gulf trial powered for the between-group adherence difference and for prespecified subgroup effects, an economic evaluation linking adherence to direct device cost and downstream HbA_1c_-attributable health care use, and an implementation study examining clinician trust, time burden, and integration with the Al Shifa electronic record. Mechanistic work could extend the model to predict not only adherence but also time-in-range and diabetic ketoacidosis risk, conditional on adherence trajectory, while preserving the transparency standards established in the pilot.

Beyond peer-reviewed publication, dissemination will include a MoH policy brief, an Arabic-language family-facing summary, scientific presentations at ISPAD, EASD-Diabetes Technology, and Gulf Diabetes Conference meetings, and a closed clinician webinar coordinated with the national pediatric endocrinology network. A version of the OMNIdiasense protocol, model card, and data dictionary will be made available on a MoH page subject to information-governance review.

### Conclusions

Optimizing CGM use is essential to realize the clinical and economic benefits of Oman’s national pediatric T1DM initiative. If the formative and pilot phases produce the anticipated findings, this protocol would generate a locally developed, ethically governed AI-plus-MI care pathway that is feasibility-tested and ready to be evaluated in a definitive multisite trial. At this stage of the program, the OMNIdiasense tool is not recommended for clinical decision-making outside the studies described here, and any future scale-up is conditional on satisfactory external validation, demonstrated fairness, and ethics reapproval. The model is intended as decision support and not as a gatekeeping mechanism for CGM access.

## Appendix

Multimedia Appendix 1 Substudy 1 data-extraction form (Al Shifa).

Multimedia Appendix 2 STROBE checklist.

Multimedia Appendix 3 Qualitative topic guide for adolescents/parents and healthcare workers.

Multimedia Appendix 4 Sub-study 2 face-to-face interview booklet (BenCGM, BurCGM, RSQ-PD, SEDM, PAID-Peds, PAQ-A, dietary screener, and open-ended items).

Multimedia Appendix 5 COREQ checklist.

Multimedia Appendix 6 Information sheet and consent forms.

Multimedia Appendix 7 OMNIdiasense model card, including intended use, training data summary, performance by subgroup, calibration, limitations, and decommissioning conditions.

Multimedia Appendix 8 Motivational interviewing–consultation and rubric.

Multimedia Appendix 9 Satisfaction exit surveys (parents and healthcare workers).

Multimedia Appendix 10 SPIRIT 2025 checklist.

Multimedia Appendix 11 Ethical approval.

Multimedia Appendix 12 Grant award letter (RIA/SRP/MoH/25/01).

## Abbreviations

BenCGM: Benefits of Continuous Glucose Monitoring Scale
BurCGM: Burden of Continuous Glucose Monitoring Scale
CGM: continuous glucose monitoring
CONSORT: Consolidated Standards of Reporting Trials
COREQ: Consolidated Criteria for Reporting Qualitative Research
DMC: data monitoring committee
EASD: European Association for the Study of Diabetes
HbA_1c_: hemoglobin A_1c_
ISPAD: International Society for Pediatric and Adolescent Diabetes
ISRCTN: International Standard Randomized Controlled Trial Number
LFT: liver function test
MI: motivational interviewing
MoH: Ministry of Health
PAID-Peds: Problem Areas in Diabetes-Pediatric version
PAQ-A: Physical Activity Questionnaire for Adolescents
RCT: randomized controlled trial
RFT: renal function tests
RM: research manager
RSQ-PD: Response to Stress Questionnaire-Pediatric Diabetes
SEDM: Self-Efficacy for Diabetes Management Scale
SHAP: Shapley additive explanations
SPIRIT: Standard Protocol Items: Recommendations for Interventional Trials
STROBE: Strengthening the Reporting of Observational Studies in Epidemiology
T1DM: type 1 diabetes mellitus
TRIPOD+AI: Transparent Reporting of a Multivariable Prediction Model for Individual Prognosis or Diagnosis + Artificial Intelligence

## Data Availability

Deidentified (pseudonymized) datasets generated and/or analyzed during this study will be made available by the corresponding author upon reasonable request, subject to approval by the Oman Ministry of Health and in accordance with applicable ethical and data governance requirements.
