# Supplementary material for: An AI-Assisted Tool to Predict Continuous Glucose Monitor Adherence in Children With Type 1 Diabetes in Oman: Protocol for a Multiphase Mixed Methods Translational Study
Source: JMIR Res Protoc. 2026 Jul 13;15:e99626. doi: 10.2196/99626 (PMC13408470; doi:10.2196/99626)
Supplement: Multimedia Appendix 1 [file resprot_v15i1e99626_app1.pdf]

Region	Patient ID	Age	Gender	Region	Health Facility	Clinic	ht (Before C	ht (Before C

I (Before C[illegible]	Date of result	After 3 to 6 months			Date of result	
		Weight (After CGM)	[illegible]ght (After C[illegible]	BMI (After CGM)		Age at Diagno[illegible]

Answer with YES / NO

ation of Dia	ent(s) with	ling(s) with	Hypertensio	Dyslipidemi	yroid Disord	ovascular D	athy/Nephr	eliac Diseas

	Date of issu	Item Code	nsulin Befor	Item Code	nsulin Befor	Item Code	dication before CGM	
Other Comorbidity								Date of issu

	List medication changes after receiving CGM						Blood Pressure (Befo	Rate (Before
Item Code	nsulin Afte	Item Code	nsulin Befor	Item Code	edication Af	Date of issue		

ressure (Aft	Rate (After	1c (Before C	erol (Before	T (Before CC	(Before CC	R (Before C	1c (After C	terol (After

T (After CG	T (After CG	R (After C(	ate of the te	f visit before	VISIT AFT	M Device T	of Device (C	CGM Devic

bles Usage I	tion of CGM	ow-up Visits	erse Events	Patient Compliance with CGM	Additional Comments
