# Supplementary material for: An AI-Assisted Tool to Predict Continuous Glucose Monitor Adherence in Children With Type 1 Diabetes in Oman: Protocol for a Multiphase Mixed Methods Translational Study
Source: JMIR Res Protoc. 2026 Jul 13;15:e99626. doi: 10.2196/99626 (PMC13408470; doi:10.2196/99626)
Supplement: Multimedia Appendix 2 [file resprot_v15i1e99626_app2.pdf]

Critical Appraisal & Supplementary Materials

Protocol appraised: *An Artificial Intelligence-Assisted Tool to Predict Continuous Glucose Monitor Adherence in Children with Type 1 Diabetes in Oman: Protocol for a Multi-Phase Mixed Methods Translational Study* (JMIR Research Protocols submission, April 2026; ISRCTN15827616).

2. STROBE Checklist (cross-sectional + cohort components)

STROBE applies to sub-study 1 (retrospective cohort) and the quantitative arm of sub-study 2 (cross-sectional). Each item is appraised once against the protocol; where reporting differs by sub-study, this is noted.

#	STROBE item	Description	Reported?	Page(s)
**Title and abstract**				
1a	Title/abstract — design	Indicate the study's design with a commonly used term in the title or the abstract	Yes ("retrospective cohort", "cross-sectional mixed methods" in abstract)	p. 1-2
1b	Title/abstract — informative summary	Informative and balanced summary of what was done and what was found	Yes (structured BOMRC abstract)	p. 1-2
**Introduction**				
2	Background/rationale	Scientific background and rationale for the investigation	Yes	p. 3-5
3	Objectives	Specific objectives, including any prespecified hypotheses	Yes	p. 4-5

**Methods**				
4	Study design	Present key elements of study design early in the paper	Yes	p. 5; sub-study 1<br>p. 5; sub-study 2<br>p. 6
5	Setting	Describe the setting, locations, and relevant dates, including periods of recruitment, exposure, follow-up, and data collection	Yes (11 governorates; July 2024–February 2025 for cohort; June 2026 onward for sub-study 2)	p. 5; p. 6
6a	Participants — eligibility (cohort)	Give the eligibility criteria, and the sources and methods of selection of participants; describe methods of follow-up	Yes (all CGM recipients July 2024–Feb 2025; ≥3 months follow-up window)	p. 5
6a	Participants — eligibility (cross-sectional)	Give the eligibility criteria, and the sources and methods of selection of participants	Yes (random sample 10–18 y from sub-study 1 cohort)	p. 6
6b	Participants — matched studies	For matched studies, give matching criteria and number of exposed/unexposed	Not applicable	—
7	Variables	Clearly define all outcomes,	Yes (variables listed;	p. 5-6; p. 7

		exposures, predictors, potential confounders, and effect modifiers; give diagnostic criteria, if applicable	Optimizer/Sub-user dichotomy defined)	
8	Data sources / measurement	For each variable of interest, give sources of data and details of methods of assessment (measurement); describe comparability of assessment methods if there is more than one group	Yes (Al Shifa HIS extraction; validated instruments BenCGM, BurCGM, RSQ, SEDM, PAID-Peds, PAQ-A)	p. 5-6; p. 7
9	Bias	Describe any efforts to address potential sources of bias	Partial — randomization in sub-study 2 sampling and blinded outcome assessment in sub-study 3 reduce bias; recall and social-desirability bias acknowledged in Limitations only	p. 7; p. 12 (Limitations)
10	Study size	Explain how the study size was arrived at	Yes (≥10 events per variable, 240 minimum, governorate-stratified)	p. 7

11	Quantitative variables	Explain how quantitative variables were handled in the analyses; if applicable, describe which groupings were chosen and why	Yes (CGM days/week dichotomised at 6/7; HbA1c continuous; descriptive statistics; pre-post comparisons)	p. 6; p. 7
12a	Statistical methods — main	Describe all statistical methods, including those used to control for confounding	Yes (paired t/Wilcoxon, McNemar, chi-square, ANOVA, multivariable logistic)	p. 6; p. 7
12b	Statistical methods — subgroups	Describe any methods used to examine subgroups and interactions	Partial — governorate comparison planned; formal interaction testing not specified	p. 6
12c	Statistical methods — missing data	Explain how missing data were addressed	Partial — multiple imputation described for the RCT; cohort missing-data approach not specified	p. 10
12d	Statistical methods — sampling	Cohort study: explain how loss to follow-up was addressed; cross-sectional: describe analytical methods taking account of sampling strategy	Partial — sub-study 1 follow-up rule (≥3 months) stated; loss-to-follow-up handling not specified; sub-study 2 stratification described	p. 5; p. 7

12e	Sensitivity analyses	Describe any sensitivity analyses	Partial — sensitivity analyses for missing data in RCT only	p. 10
**Results**				
13a-c	Participants	Numbers at each stage; reasons for non-participation; consider flow diagram	Not applicable (protocol — results forthcoming; flow planned in Figure 1)	—
14a-c	Descriptive data	Characteristics of study participants; missing data; follow-up time	Not applicable (protocol)	—
15	Outcome data	Number of outcome events or summary measures over time	Not applicable (protocol)	—
16a-c	Main results	Unadjusted and confounder-adjusted estimates with precision; category boundaries; translation of relative to absolute risk	Not applicable (protocol)	—
17	Other analyses	Other analyses done — e.g., analyses of subgroups and interactions, and sensitivity analyses	Not applicable (protocol)	—
**Discussion**				

18	Key results	Summarise key results with reference to study objectives	Yes (anticipated principal findings)	p. 11
19	Limitations	Discuss limitations, taking into account sources of potential bias or imprecision; discuss both direction and magnitude of any potential bias	Yes	p. 12
20	Interpretation	Cautious overall interpretation considering objectives, limitations, multiplicity of analyses, results from similar studies, and other relevant evidence	Yes	p. 11 (Comparison With Prior Work); p. 12
21	Generalisability	Discuss the generalisability (external validity) of the study results	Partial — single-country generalisability noted	p. 12
**Other information**				
22	Funding	Give the source of funding and the role of the funders for the present study and, if applicable, for the original study on which	Yes	p. 11; p. 12

		the present article is based		

STROBE summary. All applicable methodological items are reported or partially reported. Result-section items 13-17 are correctly *not applicable* at the protocol stage. Recommended pre-submission strengthening: add a brief bias-mitigation paragraph to sub-studies 1 and 2 (item 9), and clarify cohort missing-data handling and loss-to-follow-up (items 12c and 12d).
