# Supplementary material for: An AI-Assisted Tool to Predict Continuous Glucose Monitor Adherence in Children With Type 1 Diabetes in Oman: Protocol for a Multiphase Mixed Methods Translational Study
Source: JMIR Res Protoc. 2026 Jul 13;15:e99626. doi: 10.2196/99626 (PMC13408470; doi:10.2196/99626)
Supplement: Multimedia Appendix 3 [file resprot_v15i1e99626_app3.docx]

# Qualitative Topic Guide (Multimedia Appendix 5)

A single, stratified topic guide is provided to harmonise the open-ended component of sub-study 2 (parents/caregivers and adolescents) with the qualitative satisfaction component of sub-study 3 (parents/caregivers and healthcare workers). Two parallel versions share the same architecture so that responses can be triangulated; cultural framing, vocabulary, and probes differ where appropriate.

## 4.1 Operational notes for interviewers

- **Format.** Semi-structured, face-to-face, in Arabic or English at the participant’s preference. Where adolescents and caregivers participate together, the interviewer briefly interviews each separately to reduce cross-influence.
- **Duration.** 30–45 minutes per interview.
- **Recording.** Audio-recorded with explicit consent; verbatim transcription into the language of the interview, then forward translation to English by a bilingual researcher and back-translation check.
- **Field notes.** Contemporaneous notes capturing tone, body language, interruptions, and reflexive observations.
- **Member checking.** Summary of key themes returned to a 20% subsample for comment.
- **Cultural sensitivity.** Female interviewers offered to female caregivers and adolescents wherever requested; interviews conducted in private, gender-respectful spaces; questions about religious or family norms framed neutrally.

## 4.2 Topic guide — Adolescents and parents/caregivers (sub-study 2 + sub-study 3 follow-up)

### Opening (rapport-building, 3–5 min)

1. Thank you for agreeing to talk with me today. To start, could you tell me a little about your child / yourself — age, school year, who lives at home with you?
2. When and how was the diabetes first diagnosed? What did that time feel like for the family?

### Section A — Living with type 1 diabetes

1. Walk me through a typical day with the diabetes — from waking up, through school/work, until bedtime.
2. What is the most difficult part of looking after the diabetes day-to-day?
3. What helps you cope on hard days? *Probes:* family support, school staff, faith and community, healthcare team.

### Section B — Experience with the continuous glucose monitor (CGM)

1. Tell me about the day you/your child received the CGM. What did you understand about it?
2. How is wearing the CGM going now? *Probes:* comfort, skin reactions, sensor falling off, alarms.
3. How often do you wear the sensor? On the days when you don’t wear it, what is going on?
4. What benefits has the CGM brought, if any? *Probes:* fewer fingerpricks, school freedom, parental peace of mind, sleep, sport.
5. What burdens or downsides has the CGM brought? *Probes:* alarm fatigue, cost, body image, social comments, technology stress.
6. Has the CGM changed how you and your child / you and your parents talk about diabetes?

### Section C — School, friends, and physical activity

1. How does the school handle the CGM and diabetes more generally? *Probes:* teachers, sports lessons, PE exemptions, exam accommodations.
2. Do friends know about the CGM? How have they reacted?
3. How has the CGM affected physical activity, sport, and play?

### Section D — Diet and lifestyle

1. Tell me about meals at home and outside the home. How does the CGM influence food choices?
2. Are there foods, meals, or occasions where the CGM is particularly hard to use? *Probes:* Eid feasts, fasting practices, weddings, sleepovers.

### Section E — Healthcare team and the AI-assisted tool (sub-study 3 follow-up only)

1. After you started working with the new AI-assisted tool (OMNIdiasense), what did you think of it? *Probes:* clarity, language, time taken, trust in its predictions.
2. Did the tool change anything about the way you use the CGM? In what way?
3. The motivational interviewing sessions — what was helpful and what was not?
4. If a family in your neighbourhood was about to receive a CGM, what would you tell them about the tool and the sessions?

### Section F — Hopes, fears, and recommendations

1. Looking ahead one year, what do you hope will be different about your child’s diabetes care?
2. If the Ministry of Health were sitting here today, what one change would make the biggest difference?
3. Is there anything important about your experience that we have not yet talked about?

### Closing (2 min)

- Thank participant; explain next steps and how findings will be used; remind of contact for questions; provide leaflet on local support resources.

## 4.3 Topic guide — Healthcare workers (sub-study 3 satisfaction component)

Apply same operational notes (4.1).

### Opening

1. Could you start by telling me about your role and how long you have worked with children with type 1 diabetes?

### Section A — Pre-OMNIdiasense practice

1. Before this project, how did you decide which children would benefit most from CGM, and how to support them after they received it?
2. What were the biggest day-to-day challenges in supporting CGM use in your clinic?

### Section B — Using the OMNIdiasense tool

1. Walk me through how you use the OMNIdiasense tool with a new patient. *Probes:* time taken, integration with Al Shifa, ease of explanation to families.
2. How accurate do the predictions feel against your clinical judgement?
3. When the tool flags a likely sub-user, how do you act on that information?
4. What does the tool do well? Where does it fall short?

### Section C — Motivational interviewing skills

1. How has the MI training changed your conversations with families?
2. Where are you most confident applying MI? Where do you still feel uncertain?

### Section D — Service delivery, equity, and culture

1. Are there families for whom the tool seems to work especially well, or especially poorly?
2. Are there cultural, linguistic, or geographic considerations the tool doesn’t capture?
3. Does the tool change patient flow, dispensing pathways, or stock management at your facility?

### Section E — Safety, ethics, and trust

1. Have you experienced situations where the tool’s recommendation conflicted with clinical safety? How did you resolve it?
2. What concerns, if any, do you have about data privacy and consent?

### Section F — Sustainability and scale-up

1. If OMNIdiasense were rolled out nationally tomorrow, what would need to change at facility, regional, and ministry levels?
2. What would help you sustain the practice changes after the project ends?

### Closing

- Thank participant; offer summary of aggregated findings; confirm member-checking opportunity.
