# Supplementary material for: An AI-Assisted Tool to Predict Continuous Glucose Monitor Adherence in Children With Type 1 Diabetes in Oman: Protocol for a Multiphase Mixed Methods Translational Study
Source: JMIR Res Protoc. 2026 Jul 13;15:e99626. doi: 10.2196/99626 (PMC13408470; doi:10.2196/99626)
Supplement: Multimedia Appendix 4 [file resprot_v15i1e99626_app4.pdf]

OMNIdiasense Research Questionnaire Booklet

Longitudinal Visits (Baseline, 3, 6, and 12 Months)

OMNIdiasense Research Questionnaire Booklet

Study Title: Correlates of Continuous Use of CGMs in Children with T1DM in Oman (OMNIdiasense)

Trial Registration: ISRCTN15827616 | **Ethics:** MoH/CSR/24/29506

Population: Adolescents aged 10–18 years with Type 1 Diabetes (current or past CGM users)

Mode: Interviewer-administered, face-to-face

Visit schedule: Baseline → 3 months → 6 months → 12 months

Participant ID & Visit Tracker

Participant ID	____________________
Initials	____________________
Site / Clinic	____________________
Group allocation	☐ Intervention ☐ Control ☐ Single-arm

Visit	Target window	Date completed	Interviewer	Duration
Baseline (V0)	Day 0	__ /__ /____	__________	__ min
3-month (V1)	Day 90 ± 14	__ /__ /____	__________	__ min
6-month (V2)	Day 180 ± 14	__ /__ /____	__________	__ min
12-month (V3)	Day 365 ± 28	__ /__ /____	__________	__ min

How to Use This Booklet

This booklet is divided into three parts:

1. **Schedule of Assessments** what is collected at each visit (one-page overview).
2. **Visit Forms** one short form per visit (Baseline, 3-month, 6-month, 12-month). Use only the form for the current visit.
3. **Instruments Library** full text of all questionnaires, printed **once**. Visit forms direct you to the relevant instrument and tell you to record responses on that instrument's response grid.

Each instrument has a **response grid** at the end of its section with a column for every visit at which it is administered. Tick the appropriate column.

PART 1 — Schedule of Assessments

Domain / Instrument	Baseline	3 mo	6 mo	12 mo
Eligibility & assent / consent	●	—	—	—
Demographics	●	—	—	—
Clinical measures (HbA1c, weight, height, BP)	●	●	●	●
CGM-use classification (days/week worn)	●	●	●	●
Brief check-in (3-month touchpoint only)	—	●	—	—
BenCGM — Benefits of CGM (8 items)	●	—	●	●
BurCGM — Burden of CGM (8 items)	●	—	●	●
SEDM — Self-Efficacy for Diabetes Management (10)	●	●	●	●
PAID-Peds — Diabetes Distress (20 items)	●	—	●	●
PAQ-A — Physical Activity (8 items, 7-day recall)	●	—	●	●
Short Dietary Screener (8 items)	●	—	●	●
RSQ-PD — Coping & Stress (10 stressors + 57 coping)	●	—	—	●
Open-ended CGM experience (qualitative)	●	—	—	●
Visit completion notes	●	●	●	●

● = administer at this visit | — = not administered

Estimated visit duration: Baseline ≈ 60 min · 3-month ≈ 15 min · 6-month ≈ 35 min · 12-month ≈ 60 min.

PART 2 Visit Forms

Visit 0 Baseline

Target: Day 0 (enrolment) | **Duration:** ~60 minutes

B-1. Eligibility & Consent

B-1.1 Age (10–18 inclusive): years ☐ Eligible ☐ Not eligible STOP

B-1.2 Confirmed diagnosis of T1DM (Al-Shifa record reviewed)? ☐ Yes ☐ No STOP

B-1.3 CGM history? ☐ Currently uses CGM ☐ Past CGM user, stopped ☐ Never STOP

B-1.4 Able to complete interview today? ☐ Yes ☐ No defer

B-1.5 — Assent / Consent

Read aloud: “This study is about your experience using a CGM device. Your answers will help us better support young people with diabetes. All information is private and will not affect your care. Participation is voluntary; you may stop at any time.”

☐ Verbal assent obtained from participant ☐ Written informed consent obtained from parent/guardian (required <18 y) ☐ Signed consent form filed in study TMF

Interviewer signature: ____________________ Date: __ /__ /____

B-2. Demographics — *administer once at baseline only*

B-2.1 Age: _____ years **B-2.2** Sex: ☐ Male ☐ Female ☐ Prefer not to say

B-2.3 Region of residence: ☐ Muscat ☐ Dhofar ☐ Musandam ☐ Al Buraimi ☐ Al Dakhiliyah ☐ Al Batinah North ☐ Al Batinah South ☐ Al Sharqiyah North ☐ Al Sharqiyah South ☐ Al Dhahirah ☐ Al Wusta ☐ Other: __________

B-2.4 Insulin therapy: ☐ MDI ☐ Pump (CSII) ☐ Both ☐ Other: ________

B-2.5 Duration of T1DM (from Al-Shifa): _____ years

B-2.6 Highest school grade attained: __________

B-2.7 Primary caregiver: ☐ Mother ☐ Father ☐ Both ☐ Other: ________

B-3. Clinical Measures

**Measure**	**Value**	**Unit / note**
HbA1c (most recent, ≤30 days)	_______	% / mmol/mol
Date of HbA1c	__ /__ /____	
Weight	_______	kg
Height	_______	cm
BMI (calculated)	_______	kg/m^2
BMI z-score (WHO)	_______	
Systolic BP	_______	mmHg
Diastolic BP	_______	mmHg

B-4. CGM-Use Classification

B-4.1 Days/week wearing CGM in past month: ☐ 0 ☐ 1–2 ☐ 3–5 ☐ 6–7

B-4.2 Completely stopped using CGM? ☐ Yes ☐ No

B-4.3 Classification: ☐ CGM Optimizer (≥6 days/wk) ☐ CGM Sub-user (<6 days/wk or stopped)

B-5. Questionnaires — *administer in this order; record on the response grid in Part 3*

1. ☐ **BenCGM** (Library -A) 8 items

2. ☐ **BurCGM** (Library -B) 8 items
3. ☐ **SEDM** (Library -C) 10 items
4. ☐ **PAID-Peds** (Library -D) 20 items
5. ☐ **PAQ-A** (Library -E) 8 items
6. ☐ **Short Dietary Screener** (Library -F) 8 items
7. ☐ **RSQ-PD** (Library -G) 10 stressors + 57 coping items
8. ☐ **Open-ended CGM experience** (Library -H) 11 items

B-6. Visit Completion Notes (Baseline)

Interviewer: ___________ Date: __ /__ /____ Duration: ____ min

Engagement: ☐ Very engaged ☐ Moderate ☐ Required prompting ☐ Declined items

Sections skipped / incomplete: ____

Observations: ____

Visit 1 3 Months

Target: Day 90 ± 14 | **Duration:** ~15 minutes

M3-1. Clinical Measures

Measure	Value	Unit
HbA1c (most recent ≤30 days)	________	% / mmol/mol
Date of HbA1c	__ /__ /____	
Weight	________	kg
Height	________	cm
BMI	________	kg/m²
Systolic / Diastolic BP	___ / ___	mmHg

M3-2. CGM-Use Classification

M3-2.1 Days/week wearing CGM in past month: ☐ 0 ☐ 1–2 ☐ 3–5 ☐ 6–7

M3-2.2 Completely stopped using CGM? ☐ Yes ☐ No

M3-2.3 Classification: ☐ Optimizer ☐ Sub-user

M3-3. Brief Check-in (3-month touchpoint)

M3-3.1 Since baseline, has your CGM use changed? ☐ Increased ☐ Stayed the same ☐ Decreased ☐ Stopped

M3-3.2 In the past month, what was the **single biggest** facilitator of CGM use?

____

M3-3.3 In the past month, what was the **single biggest** barrier to CGM use?

____

M3-3.4 Any device problems since last visit (skin, alarms, sensor failure, supply)? ☐ No ☐ Yes — describe: ____

M3-3.5 Any serious adverse events (DKA, severe hypoglycaemia, hospitalisation)? ☐ No ☐ Yes — complete SAE form and notify PI within 24 h

M3-4. SEDM administer*; record on Library -C grid (3-mo column)*

☐ SEDM (10 items) administered

M3-5. Visit Completion Notes (3 months)

Interviewer: __________ Date: __ /__ /____ Duration: ____ min

Engagement: ☐ Very ☐ Moderate ☐ Required prompting ☐ Declined

Notes: ____

Visit 2 -6 Months

Target: Day 180 ± 14 | **Duration:** ~35 minutes

M6-1. Clinical Measures

Measure	Value	Unit
HbA1c (most recent ≤30 days)	_______	% / mmol/mol
Date of HbA1c	__ /__ /____	
Weight	_______	kg
Height	_______	cm
BMI	_______	kg/m²
Systolic / Diastolic BP	___ / ___	mmHg

M6-2. CGM-Use Classification

M6-2.1 Days/week wearing CGM in past month: ☐ 0 ☐ 1–2 ☐ 3–5 ☐ 6–7

M6-2.2 Completely stopped using CGM? ☐ Yes ☐ No

M6-2.3 Classification: ☐ Optimizer ☐ Sub-user

M6-3. Questionnaires

1. ☐ **BenCGM** (Library -A) 8 items
2. ☐ **BurCGM** (Library -B) 8 items
3. ☐ **SEDM** (Library -C) 10 items
4. ☐ **PAID-Peds** (Library -D) 20 items

5. ☐ **PAQ-A** (Library §E) 8 items
6. ☐ **Short Dietary Screener** (Library §F) 8 items

M6-4. Adverse Events Since Last Visit

☐ No events ☐ DKA ☐ Severe hypoglycaemia ☐ Hospitalisation ☐ Skin reaction ☐ Other: _______

Details: ____

M6-5. Visit Completion Notes (6 months)

Interviewer: __________ Date: __ /__ /____ Duration: ____ min

Engagement: ☐ Very ☐ Moderate ☐ Required prompting ☐ Declined

Notes: ____

Visit 3 -12 Months (Final Visit)

Target: Day 365 ± 28 | **Duration:** ~60 minutes

M12-1. Clinical Measures

Measure	Value	Unit
HbA1c (most recent ≤30 days)	_______	% / mmol/mol
Date of HbA1c	__ /__ /____	
Weight	_______	kg
Height	_______	cm
BMI	_______	kg/m²
Systolic / Diastolic BP	___ / ___	mmHg

M12-2. CGM-Use Classification

M12-2.1 Days/week wearing CGM in past month: ☐ 0 ☐ 1–2 ☐ 3–5 ☐ 6–7

M12-2.2 Completely stopped using CGM? ☐ Yes ☐ No

M12-2.3 Classification: ☐ Optimizer ☐ Sub-user

M12-3. Questionnaires

1. ☐ **BenCGM** (Library -A) 8 items
2. ☐ **BurCGM** (Library -B) 8 items
3. ☐ **SEDM** (Library -C) 10 items
4. ☐ **PAID-Peds** (Library -D) 20 items
5. ☐ **PAQ-A** (Library -E) 8 items
6. ☐ **Short Dietary Screener** (Library -F) 8 items

7. ☐ **RSQ-PD** (Library G) full
8. ☐ **Open-ended CGM experience** (Library H) 11 items

M12-4. Adverse Events Since Last Visit

☐ No events ☐ DKA ☐ Severe hypoglycaemia ☐ Hospitalisation ☐ Skin reaction ☐ Other:

Details: ____

M12-5. Study Exit

☐ Completed all visits ☐ Withdrew (reason: ___________) ☐ Lost to follow-up

Final remarks: ____

M12-6. Visit Completion Notes (12 months)

Interviewer: __________ Date: __ /__ /____ Duration: ____ min

PART 3 Instruments Library

Each instrument is printed **once**. The response grid at the end of every instrument has a column for each visit at which it is administered. Tick the appropriate column.

A. BenCGM Benefits of CGM (8 items)

Validated 8-item scale of perceived CGM benefits in youth (12–19 y). 5-point Likert.

Instructions: *"Please rate how much you agree with each statement about your CGM."*

Response scale: 1 = Strongly disagree · 2 = Disagree · 3 = Neutral · 4 = Agree · 5 = Strongly agree

	Item	BL	6M	12M
A.1	My CGM helps me manage my blood sugar better.			
A.2	My CGM gives me peace of mind about my diabetes.			
A.3	My CGM helps me make better decisions about food.			
A.4	My CGM helps me make better decisions about activity.			
A.5	My CGM helps me catch lows before they get serious.			
A.6	My CGM helps me sleep better.			
A.7	My CGM has improved my overall diabetes control.			
A.8	I would recommend a CGM to other young people.			
	**Total (sum, 8–40) — higher = greater benefit**			

B. BurCGM Burden of CGM (8 items)

Validated 8-item scale of perceived CGM burden in youth. 5-point Likert.

Instructions: *"Please rate how much you agree with each statement about wearing your CGM."*

Response scale: 1 = Strongly disagree · 2 = Disagree · 3 = Neutral · 4 = Agree · 5 = Strongly agree

	**Item**	**BL**	**6M**	**12M**
B.1	Wearing my CGM is uncomfortable.			
B.2	I feel embarrassed about others seeing my CGM.			
B.3	The CGM alarms bother or annoy me.			
B.4	Inserting / changing the sensor is painful or stressful.			
B.5	The CGM gets in the way of activities I want to do.			
B.6	My skin gets irritated by the CGM.			
B.7	I worry about the CGM sticking out or being knocked off.			
B.8	The CGM makes me think about my diabetes too much.			
	**Total (sum, 8–40) — higher = greater burden**			

C. SEDM Self-Efficacy for Diabetes Management (10 items)

Validated 10-item scale; 1–10 confidence rating per item.

Instructions: *"On a scale from 1 (not at all confident) to 10 (completely confident), how confident are you that you can…"*

	**Item**	**BL**	**3M**	**6M**	**12M**
C.1	…check your blood sugar as often as needed?				
C.2	…adjust your insulin based on your blood sugar?				
C.3	…count carbohydrates correctly?				
C.4	…recognise and treat low blood sugar?				
C.5	…recognise and treat high blood sugar?				
C.6	…take care of your diabetes when away from home?				
C.7	…take care of your diabetes when you are sick?				
C.8	…handle your diabetes when you feel stressed?				
C.9	…explain your diabetes to friends or teachers?				
C.10	…keep wearing your CGM consistently every day?				
	**Total (sum, 10–100) — higher = greater efficacy**				

D. PAID-Peds Diabetes Distress (20 items)

Validated 20-item Problem Areas in Diabetes Pediatric scale. 0–4 scale, reverse-scored, summed and ×1.25 to give 0–100 (higher = greater distress).

Instructions: *"How much of a problem has each of these been for you in the past month?"*

Response scale: 0 = Not a problem · 1 = Minor problem · 2 = Moderate problem · 3 = Somewhat serious problem · 4 = Serious problem

	**Item**	**BL**	**6M**	**12M**
D.1	Feeling scared about living with diabetes.			
D.2	Feeling sad about having diabetes.			
D.3	Feeling angry about having diabetes.			
D.4	Worrying about low blood sugars.			
D.5	Worrying about high blood sugars.			
D.6	Worrying about long-term complications.			
D.7	Feeling "burned out" by the daily work of diabetes.			
D.8	Feeling alone with my diabetes.			
D.9	Feeling friends don't understand my diabetes.			
D.10	Feeling family doesn't understand my diabetes.			
D.11	Arguing with parents about diabetes care.			
D.12	Feeling I can never take a break from diabetes.			
D.13	Feeling overwhelmed by daily diabetes care.			
D.14	Feeling that diabetes controls my life.			
D.15	Feeling unmotivated to manage my diabetes.			
D.16	Feeling guilty when I don't manage diabetes well.			
D.17	Feeling that diabetes makes me different.			
D.18	Worrying about weight changes from diabetes.			
D.19	Worrying about eating in front of others.			
D.20	Feeling embarrassed about having diabetes.			
	**Sum (0–80) → ×1.25 → Total score (0–100)**			

E. PAQ-A Physical Activity Questionnaire for Adolescents (8 items)

> *Validated 7-day recall (excludes today). Items rated 1-5; total = mean of items E.1-E.7. Item E.8 is a validity flag.*

Instructions: *"I am going to ask you about the physical activities you did over the past 7 days (not including today)."*

Scale: 1 = No / very little activity · 2 = Slightly active · 3 = Moderately active · 4 = Very active · 5 = Extremely active

	Item	BL	6M	12M
E.1	Spare-time sport / exercise (any kind)			
E.2	Activity during PE class			
E.3	Activity during recess / lunch break			
E.4	Activity right after school			
E.5	Activity in the evening			
E.6	Activity on the weekend			
E.7	Overall activity in the past 7 days			
E.8	**Validity flag:** Sick / injured this week? (Y / N)			
	**PAQ-A score = mean of E.1–E.7 (exclude E.8)**			

F. Short Dietary Screener (8 items)

Brief dietary screener relevant to T1DM management.

Instructions: *"Think about your usual eating habits in the past week."*

Response keys (record the numeric code in BL / 6M / 12M columns):

- **F.1, F.2 (servings/day):** 0 = None · 1 = 1/day · 2 = 2/day · 3 = ≥3/day
- **F.3 (sugary drinks):** 0 = Never · 1 = 1–2/wk · 2 = 3–5/wk · 3 = 1×/day · 4 = >1×/day
- **F.4, F.5 (fast food, snacks):** 0 = Never · 1 = 1–2/wk · 2 = 3–5/wk · 3 = ≥1×/day
- **F.6 (main meals/day):** 1 · 2 · 3 · 4 (>3)
- **F.7 (carb counting):** 0 = Never · 1 = Rarely · 2 = Sometimes · 3 = Most of the time · 4 = Always
- **F.8 (diet plan):** 0 = No · 1 = Yes · 2 = Unsure

	Item	BL	6M	12M
F.1	Servings of fruit per day			
F.2	Servings of vegetables per day			
F.3	Sugary drinks (soda, juice, energy drinks)			
F.4	Fast food (burgers, pizza, fried chicken)			
F.5	Snacks (chips, sweets, biscuits, candy)			
F.6	Main meals per day			
F.7	Carbohydrate counting			
F.8	Following a diet plan from diabetes team			

G. RSQ-PD Responses to Stress Questionnaire (Pediatric Diabetes Version)

10 stressor ratings (0–3) + 57 coping items (0–4) across 5 factors: Primary Control Engagement Coping (PCEC), Secondary Control Engagement Coping (SCEC),

Disengagement Coping (DC), Involuntary Engagement (IE), Involuntary Disengagement (ID). Administered at Baseline and 12-month only.

G.1 Stressor Ratings (10 items, 0 = not at all stressful · 3 = very stressful)

	Stressor	BL	12M
G.1.1	Checking blood sugar / using CGM		
G.1.2	Taking insulin (injections / pump)		
G.1.3	Counting carbohydrates		
G.1.4	Dealing with low or high blood sugar		
G.1.5	Eating with friends / at school		
G.1.6	Worrying about long-term complications		
G.1.7	Diabetes interfering with school / activities		
G.1.8	Telling friends or others about my diabetes		
G.1.9	Conflict with parents about diabetes care		
G.1.10	Feeling different from peers because of diabetes		

G.2 Coping Items (57 items, 0 = not at all · 4 = a lot)

To save space, items are grouped by factor. Tick / score each item in the BL and 12M columns.

Primary Control Engagement Coping PCEC (12 items, G.2.1–G.2.12): problem-solving, emotional expression, emotional modulation.

	Coping behaviour	BL	12M
G.2.1	Make a plan to fix the problem		
G.2.2	Try harder to solve it		
G.2.3	Get information / advice		
G.2.4	Take action steps		
G.2.5	Ask a parent / clinician for help		
G.2.6	Re-check the situation carefully		
G.2.7	Express feelings to someone trusted		
G.2.8	Talk about feelings with family		
G.2.9	Calm myself with deep breaths		
G.2.10	Listen to music / relax		
G.2.11	Use prayer / spiritual practice		
G.2.12	Other emotion-regulation strategy		

Secondary Control Engagement Coping SCEC (12 items, G.2.13–G.2.24): acceptance, cognitive restructuring, distraction, positive thinking.

	**Coping behaviour**	**BL**	**12M**
G.2.13	Accept that diabetes is part of life		
G.2.14	Look for the positive side		
G.2.15	Remind self of strengths		
G.2.16	Compare situation with worse outcomes		
G.2.17	Reframe the problem		
G.2.18	Trust things will improve		
G.2.19	Distract self with hobbies		
G.2.20	Watch TV / play games		
G.2.21	Spend time with friends		
G.2.22	Sleep / rest		
G.2.23	Exercise / physical activity		
G.2.24	Other distraction strategy		

Disengagement Coping DC (9 items, G.2.25–G.2.33): avoidance, denial, wishful thinking.

	**Coping behaviour**	**BL**	**12M**
G.2.25	Try not to think about it		
G.2.26	Avoid people who remind me of it		
G.2.27	Stay away from situations		
G.2.28	Refuse to deal with it		
G.2.29	Deny that anything is wrong		
G.2.30	Wish the problem would go away		
G.2.31	Pretend it isn't happening		
G.2.32	Imagine a different reality		
G.2.33	Other avoidance strategy		

Involuntary Engagement IE (12 items, G.2.34–G.2.45): rumination, intrusive thoughts, physiological arousal, impulsive action, emotional arousal.

	**Reaction**	**BL**	**12M**
G.2.34	Can't stop thinking about it		
G.2.35	Mind keeps replaying it		

G.2.36	Intrusive worries		
G.2.37	Worry “what if” scenarios		
G.2.38	Get angry without warning		
G.2.39	Cry without warning		
G.2.40	Rapid heartbeat / shortness of breath		
G.2.41	Stomach upset / nausea		
G.2.42	Headaches / muscle tension		
G.2.43	Feel shaky		
G.2.44	Snap or lash out		
G.2.45	Other involuntary reaction		

Involuntary Disengagement ID (12 items, G.2.46–G.2.57): emotional numbing, cognitive interference, inaction, escape.

	**Reaction**	**BL**	**12M**
G.2.46	Feel numb / nothing		
G.2.47	Detached / “spaced out”		
G.2.48	Can’t focus / pay attention		
G.2.49	Forget what I was doing		
G.2.50	Lose track of time		
G.2.51	Can’t make decisions		
G.2.52	Freeze / can’t move		
G.2.53	Mind goes blank		
G.2.54	Want to escape		
G.2.55	Hide from everyone		
G.2.56	Sleep too much		
G.2.57	Other shutdown response		

Scoring: For each factor, sum the items, then divide by total RSQ score to give a proportion (controls for response bias). Report PCEC, SCEC, DC, IE, ID proportions.

H. Open-ended CGM Experience (11 items)

Qualitative section. Record verbatim or summarised responses. Administered at Baseline AND 12-month.

	**Question**
H.1	In your own words, describe how regularly you wear your CGM. What helps you wear it consistently?

H.2	If you have ever stopped wearing it or worn it less, what were the main reasons?
H.3	How do you feel about wearing the CGM in front of others (at school, with friends, in public)?
H.4	Has having diabetes and using a CGM affected your performance or concentration at school? How?
H.5	Do your teachers or school staff know about your diabetes and CGM? How do they support you?
H.6	What are the main challenges that make it difficult to use your CGM regularly?
H.7	What things help or encourage you to use your CGM regularly?
H.8	Any side effects (skin irritation, pain, alarm fatigue, device failures)?
H.9	Any technical problems with your CGM device — what happened, and how did you handle it?
H.10	What changes or improvements would help young people use their CGM more regularly?
H.11	What advice would you give to another young person starting a CGM?

Response space (use additional sheets if required):

Baseline: ____

12-month: ____

PART 4 Quick Reference

Instrument	Items	Range	Direction	Visits administered
BenCGM	8	8-40	Higher = more benefit	BL · 6M · 12M
BurCGM	8	8-40	Higher = more burden	BL · 6M · 12M
SEDM	10	10-100	Higher = more efficacy	BL · 3M · 6M · 12M
PAID-Peds	20	0-100	Higher = more distress	BL · 6M · 12M
PAQ-A	7+1	1-5 (mean)	Higher = more active	BL · 6M · 12M
Dietary screener	8	item-specific	Descriptive	BL · 6M · 12M
RSQ-PD	10 + 57	proportions	5 factor scores	BL · 12M
Open-ended	11	qualitative	Thematic analysis	BL · 12M

Booklet Workflow Checklist (Interviewer)

☐ Confirm correct visit form before starting ☐ Verify Participant ID and date on every page ☐ Use blue or black ink only — no pencil, no white-out ☐ Single-line strikethrough for corrections; initial and date ☐ Mark unanswered items “NA” with reason ☐ Sign and date the Visit Completion Notes ☐ Lock booklet in study site cabinet within 24 h ☐ Data entry into REDCap within 7 days of visit

OMNIdiasense Study | Ministry of Higher Education, Research & Innovation, Oman | *RIA/SRP/MoH/25/01,* ***Trial Registration:*** *ISRCTN15827616 |* ***Ethics:*** *MoH/CSR/24/29506,* ***Protocol version:*** *v2*
