# Supplementary material for: An AI-Assisted Tool to Predict Continuous Glucose Monitor Adherence in Children With Type 1 Diabetes in Oman: Protocol for a Multiphase Mixed Methods Translational Study
Source: JMIR Res Protoc. 2026 Jul 13;15:e99626. doi: 10.2196/99626 (PMC13408470; doi:10.2196/99626)
Supplement: Multimedia Appendix 5 [file resprot_v15i1e99626_app5.pdf]

# Critical Appraisal & Supplementary Materials

**Protocol appraised:** *An Artificial Intelligence-Assisted Tool to Predict Continuous Glucose Monitor Adherence in Children with Type 1 Diabetes in Oman: Protocol for a Multi-Phase Mixed Methods Translational Study* (JMIR Research Protocols submission, April 2026; ISRCTN15827616).

# 3. COREQ Checklist (Tong, Sainsbury & Craig, *Int J Qual Health Care* 2007)

COREQ applies to the qualitative component of sub-study 2 (open-ended items in the face-to-face interview booklet) and the planned qualitative satisfaction component of sub-study 3.

## Domain 1 Research team and reflexivity

### Personal characteristics

| # | Item | Reported? | Page(s) |
|---|---|---|---|
| 1 | Interviewer/facilitator — Which author/s conducted the interview or focus group? | Partial — diabetes nurses and Research Managers identified as data collectors; specific named interviewers not allocated | p. 5; p. 9 |
| 2 | Credentials — What were the researcher's credentials? | Partial — author-level credentials in by-line; interviewer-level not specified | p. 1 |
| 3 | Occupation — What was their occupation at the time of the study? | Partial — "diabetes nurses" and "regional pharmacists" stated for data collection roles | p. 5; p. 9 |
| 4 | Gender — Was the researcher male or female? | Not reported | — |
| 5 | Experience and training — What experience or | Partial — three-day training programme described; | p. 9 |

| | | | |
|---|---|---|---|
| | training did the researcher have? | qualitative-specific training not detailed | |

### Relationship with participants

| # | Item | Reported? | Page(s) |
|---|---|---|---|
| 6 | Relationship established — Was a relationship established prior to study commencement? | Partial — RM-family liaison via national CGM rollout implied; not formally described | p. 5 |
| 7 | Participant knowledge of the interviewer What did the participants know about the researcher? | Not reported | — |
| 8 | Interviewer characteristics What characteristics were reported about the interviewer/facilitator? (e.g., bias, assumptions, reasons and interests in the research topic) | Not reported | — |

## Domain 2 Study design

### Theoretical framework

| # | Item | Reported? | Page(s) |
|---|---|---|---|
| 9 | Methodological orientation and theory — What methodological orientation was stated to underpin the study? | Partial — convergent mixed methods design with hybrid inductive-deductive thematic analysis stated; no explicit phenomenology/grounded theory orientation | p. 7 |

## Participant selection

| # | Item | Reported? | Page(s) |
|---|---|---|---|
| 10 | Sampling How were participants selected? | Yes (random selection from sub-study 1 cohort, governorate-stratified) | p. 7 |
| 11 | Method of approach How were participants approached? | Yes (formal invitation letter, information sheet, consent/assent through RMs) | p. 9 |
| 12 | Sample size How many participants were in the study? | Yes (≥240 for sub-study 2 quantitative + open-ended) | p. 7 |
| 13 | Non-participation How many people refused to participate or dropped out? | Not applicable (protocol) | — |

## Setting

| # | Item | Reported? | Page(s) |
|---|---|---|---|
| 14 | Setting of data collection Where was the data collected? | Yes (face-to-face at diabetes clinic settings across 11 governorates) | p. 6-7 |
| 15 | Presence of non-participants Was anyone else present besides the participants and researchers? | Not reported | — |
| 16 | Description of sample What are the important characteristics of the sample? | Yes (children 10-18 y with T1DM; Optimizer/Sub-user split) | p. 6-7 |

## Data collection

| # | Item | Reported? | Page(s) |
|---|---|---|---|

| | | | |
|---|---|---|---|
| 17 | Interview guide Were questions, prompts, guides provided by the authors? Was it pilot tested? | Partial — open-ended item domains listed (compliance, school performance, barriers/facilitators, side effects, recommendations) and a pre-pilot in Muscat is described; full topic guide not in protocol | p. 7; p. 11 |
| 18 | Repeat interviews Were repeat interviews carried out? | Partial sub-study 3 includes follow-up interviews at 3, 6, 12 months | p. 9; p. 18 |
| 19 | Audio/visual recording Did the research use audio or visual recording to collect the data? | Not reported | — |
| 20 | Field notes Were field notes made during and/or after the interview or focus group? | Not reported | — |
| 21 | Duration What was the duration of the interviews or focus groups? | Not reported | — |
| 22 | Data saturation Was data saturation discussed? | Not applicable (sample size driven by quantitative model power; saturation not the primary criterion) | — |
| 23 | Transcripts returned Were transcripts returned to participants for comment and/or correction? | Not reported | — |

# Domain 3 Analysis and findings

## Data analysis

| # | Item | Reported? | Page(s) |
|---|---|---|---|
| 24 | Number of data coders How many data coders coded the data? | Partial — double coding of 20% of transcripts described; coder count not stated | p. 7 |
| 25 | Description of the coding tree Did authors provide a description of the coding tree? | Not applicable (protocol — coding tree to emerge during analysis) | — |
| 26 | Derivation of themes Were themes identified in advance or derived from the data? | Yes (hybrid inductive-deductive) | p. 7 |
| 27 | Software What software, if applicable, was used to manage the data? | Yes (NVivo) | p. 7 |
| 28 | Participant checking Did participants provide feedback on the findings? | Not reported | — |

## Reporting

| # | Item | Reported? | Page(s) |
|---|---|---|---|
| 29 | Quotations presented Were participant quotations presented to illustrate the themes/findings? Was each quotation identified? | Not applicable (protocol) | — |
| 30 | Data and findings consistent Was there | Not applicable (protocol) | — |

| | | | |
|---|---|---|---|
| | consistency between the data presented and the findings? | | |
| 31 | Clarity of major themes Were major themes clearly presented in the findings? | Not applicable (protocol) | — |
| 32 | Clarity of minor themes Is there a description of diverse cases or discussion of minor themes? | Not applicable (protocol) | — |

**COREQ summary.** Of the 32 items, 8 are *not applicable* at the protocol stage. Of the 24 applicable items, 7 are fully reported, 11 partially reported, and 6 not reported. The principal pre-submission gaps are: explicit interviewer characteristics (items 1-5, 8), audio recording and field-note plans (items 19-20), interview duration (item 21), and member checking (item 23).
