# Supplementary material for: An AI-Assisted Tool to Predict Continuous Glucose Monitor Adherence in Children With Type 1 Diabetes in Oman: Protocol for a Multiphase Mixed Methods Translational Study
Source: JMIR Res Protoc. 2026 Jul 13;15:e99626. doi: 10.2196/99626 (PMC13408470; doi:10.2196/99626)
Supplement: Multimedia Appendix 6 [file resprot_v15i1e99626_app6.pdf]

Title:
Utilizing “OMNIdiasense” an AI- assisted tool to Predict the Behavior of Children with Type 1 Diabetes for Optimal Use of Sensor Technology in Oman: A Mixed-method, Multi-phase Translational Research Project

This is an Informed Consent Form for a group of researchers from, Ministry of Health and Ministry, Sultan Qaboos University to evaluate the effectiveness of an AI predictive tool in improving the adherence to the continuous gloucose monitoring devices for children with type 1 diabetes.

This Informed Consent Form has two parts:

- Information Sheet (to share information about the study with you)
- Certificate of Consent (for signatures if you agree that your child may participate)

Part I: Information Sheet

Introduction:

Children with Type 1 diabetes (T1DM) require close monitoring and follow up through smart use of Continuous Glucose Monitors (CGMs). In February 2024, His Majesty Sultan Haitham bin Tarik issued Royal orders to help provide electronic sensors to support children with T1DM. The Ministry of Health implemented the orders in May 2024 by distributing electronic sensors to more than 1500 Omani children from February 2024 to January 2025 across all the regions to enhance diabetes management and care. This project accompanied with series of formative multi-sub studies aimed to provide the necessary data and constructs for the development of an AI assisted behavior predictive tool. Sub studies will identify the characteristics of children with T1DM who received the CGMs, factors influencing adherence to CGMs, and then inform an AI-assisted tool to predict the behavior of children with T1DM in continues use of the CGMs. The tool will then be tested (piloted) in 100 children with T1DM who are non-complaint to CGMs to evaluate its validity, reliability and effectiveness in improving adherence to using the CGMs. Additionally, a pilot study will be performed in 50 new children with T1DM where 25 children will be offered the test prior to the introduction of the CGMs vs another 25 who will follow the usual care. The two groups (intervention vs control) will be followed prospectively for one year by a diabetic nurse in polyclinics in Muscat region. Between group differences in rate of adherence to optimum use of the CGMs, metabolic and cardiovascular measures will be assessed at 6months and 12 months follow up.

Methods:

Eleven Research Managers (RMs) will be recruited across the governorates of Oman to supervise data collection, and fulfilment of project objectives in: a) data management, and b) inter and intra departmental coordination in their respected regions. An AI- assisted assessment tool will be developed based on outcomes from the series of formative sub-studies supervised by the regional Research Managers (RMs). The proposed AI-tool will be developed, and piloted by a group of computer science experts from Ministry of health and Sultan Qaboos University. Approvals for roll out will be supervised by the PI and experts from Ministry of health and Sultan Qaboos University. The phases/sub studies associated with this project are:

Sub-study 1: The characteristics of children with T1DM who received the CGMs in Oman (data from Al Shifa system). All children who received CGMS from July 2024 to February 2025 will be included (n=1500). Data on their glycemic control will be compared at least 3 months from baseline.

Sub-study 2: Correlates of adherence to Continuous Use of CGMs in Children with T1DM in Oman: A Mixed-Design Study Based on face-to-face interviews with randomly selected children of T1DM (10-18 years of age from Sub-study 1). Demographic, psycho-social, dietary and physical activity factors influencing their CGMs use will be identified. Results from this study will inform the development of a smart AI-tool that can predict the behaviour/adherence to CGMs.

Sub-study 3: Effectiveness of an AI-assisted assessment tool in predicting adherence to CGMs in children with T1DM in Muscat. This is a pilot study (Quasi-experimental single arm design) on 100 children who were non-complaint to CGMs randomly selected from sub-study 2. The AI-assessment tool will be tested prior to the re-introduction of CGMs. Interventions to improve CGMs use will be facilitated by diabetes nurses in the polyclinics. The children will be followed up for 3 and 6 months and glycemic control and adherence to CGMs will be compared from baseline (pre-post analysis). Additional 50 children will be recruited to participate in a randomized trail where 25 children will be offered the tool prior to the introduction of the CGMs vs another 25 with usual care (not exposed to the AI assisted predictive tool (Intervention group) vs another 25 children with usual care (Control group). All children will be followed prospectively for one year. Effectiveness of the tool will be assessed based on the primary outcome of between group differences in adherence to the CGMs. Secondary outcomes will include metabolic, cardiovascular, and anthropometric measures.

Ethical approvals (attachments 1-3) for the three sub-studies were issued independently by the ethical approval committee in Ministry of Health as this project is intended to be submitted for funding within the Strategic Research Program (SRP).

Expected outcomes and results:

This is the first translational two years study in Oman for children with T1DM that aims to improve service delivery through integration of lean management tools, optimize cost utilization, use of AI and machine learning methods, and capacity building activities. The ultimate outcome from this project is to deliver an Omani made AI-assisted tool that will predict behaviors prior to dispensing the CGMs. Children with sub-optimum predictions will be assessed and educated accordingly.

Conclusion:

It is expected that optimizing CGMs use in children and adolescents with T1DM will lead to improve glycemic control, optimum quality of life and overall effective use of health resources. Further studies can include adult patients with diabetes utilizing different types of wearable glucose monitor devices/insulin pumps.

Voluntary Participation:

Participating of your son/daughter is voluntary throughout the study period. This study will be conducted in health care facilities under the supervision of health care providers. You will be able to withdraw at anytime of the study if you don’t wish to continue.

We will not be sharing with you either the questions we ask nor the responses given to us to anyone. The information is confidential and will be used for research purposes.

Benefits:

There will be no immediate and direct benefit to your child or to you, but your child's participation is likely to help us find out more about how best we can help children with T1DM benefit from the CGMs for better health outcomes.

Confidentiality:

We will not be sharing information about your son or daughter outside of the research team. The information that we collect from this research project will be kept confidential. Information about your child that will be collected from the research will be put away and no-one but the researchers will be able to see it. Any information about your child will have a number on it instead of his/her name.

Sharing of Research Findings:

At the end of the study, a written report will be prepared and shared with the concerned parties. We will also publish the results in order that other interested people may learn from our research.

Right to refuse or withdraw:

You may choose not to have your child participate in this study and your child does not have to take part in this research if she/he does not wish to do so. Your child may stop participating in the discussion/interview at any time that you or she/he wish without either of you losing any of your rights for health services.

Who to Contact:

If you have any questions, you may ask them now or later, even after the study has started. If you wish to ask questions later, you may contact any of the following:

Asma Bait Ishaq: telephone number /e-mail

PART II: Certificate of Consent

Certificate of Consent

Utilizing an AI- assisted tool to Predict the Behavior of Children with Type 1 Diabetes for Optimal Use of Sensor Technology in Oman: A Multi-phase Translational Research Project

I have been asked to give consent for my daughter/son to participate in this research study which will involve her completing one interview and, if eligible, will participate in a pilot multi-component intervention to improve the use of the continuous glucose monitors I have had the opportunity to ask questions about it and any questions that I have asked have been answered to my satisfaction:

I consent voluntarily for my child to participate in study 2 and, if eligible, in study 3 as well.

I understand that there will be no interruptions with my child’s health services.

I understand that my child can withdraw from participating in the project at any time.

I understand that data is confidential and the name of my child will **Not** be exposed.

I understand that results from this study will be utilized for research purposes and published at a later stage.

Print Name of Parent or Guardian __________________

Signature of Parent of Guardian___________________

Date ___________________________

If illiterate

A literate witness must sign.

I have witnessed the accurate reading of the consent form to the parent of the potential participant, and the individual has had the opportunity to ask questions. I confirm that the individual has given consent freely.

Print name of witness_____________________ AND Thumb print of participant

Signature of witness ______________________

Date ________________________

Statement by the researcher/person taking consent for

Utilizing an AI- assisted tool to Predict the Behavior of Children with Type 1 Diabetes for Optimal Use of Sensor Technology in Oman: A Multi-phase Translational Research Project

I have accurately read out the information sheet to the parent of the potential participant, and to the best of my ability made sure that the person understands that the following will be done:

1. Face to face interview to the child with T1DM/parent using a questionnaire.

2. Eligible students will participate in study 2 and 3

3. Participation is voluntary and confidentiality of the information will be ensured.

I confirm that the parent was given an opportunity to ask questions about the study, and all the questions asked by him/her have been answered correctly and to the best of my ability. I confirm that the individual has not been pressured into giving consent, and the consent has been given freely and voluntarily.

Name of Researcher/person taking the consent________________________

Signature

Date
