# Supplementary material for: An AI-Assisted Tool to Predict Continuous Glucose Monitor Adherence in Children With Type 1 Diabetes in Oman: Protocol for a Multiphase Mixed Methods Translational Study
Source: JMIR Res Protoc. 2026 Jul 13;15:e99626. doi: 10.2196/99626 (PMC13408470; doi:10.2196/99626)
Supplement: Multimedia Appendix 7 [file resprot_v15i1e99626_app7.pdf]

An Artificial Intelligence-Assisted Tool to Predict Continuous Glucose Monitor Adherence in Children with Type 1 Diabetes in Oman: Protocol for a Multi-Phase Mixed Methods Translational Study

Trial registration: ISRCTN15827616. Ethics: MoH/CSR/24/29506. Funding: RIA/SRP/MoH/25/01.

Multimedia Appendix 8. OMNIdiasense Model Card

This model card follows the structure recommended by Mitchell et al (2019) and the model-facts label approach of Sendak et al (2020), adapted for a clinical prediction model that has not yet been trained on real data. Sections marked Planned describe what will be reported once the model is locked. Sections marked Pre-specified describe decisions made in the protocol before any data exposure.

1. Model details

Item	Description
Model name	OMNIdiasense (Omani Diabetes Sensor Adherence Engine)
Model version	v0 protocol stage, not yet trained on real data.
Model owner	Ministry of Health, Sultanate of Oman, Directorate General of Health Services (clinical owner) and Department of Computer Science, Sultan Qaboos University (technical owner).
Contact	Thamra Al Ghafri (Thamra74@yahoo.com).
Funding	Ministry of Higher Education, Research and Innovation, Strategic Research Program (RIA/SRP/MoH/25/01).
Ethics approval	MoH/CSR/24/29506.
Trial registration	ISRCTN15827616.
Reporting standard	TRIPOD-AI (Collins et al, BMJ 2024).
Licence	Not for commercial use. Use restricted to the studies described in the protocol until external validation is complete.
Model type	Supervised binary classifier (planned candidate models: penalised logistic regression, gradient-boosted trees, support vector machines, and a small feed-forward neural network). Final model will be selected on the basis of discrimination, calibration, and interpretability.

2. Intended use

Primary intended use: to estimate, before CGM dispensing, the probability that a child aged 10–18 years with type 1 diabetes in Oman will become a CGM Sub-user (< 6 days/week of sensor wear, or discontinuation) during the first 3 months of CGM use.

Primary intended users: trained diabetes nurses and paediatric endocrinologists working in Ministry of Health diabetes clinics in Oman, supported by the Research Manager network during the pilot phase.

Intended deployment setting: outpatient paediatric diabetes clinics. The model is to be used as a decision-support adjunct integrated into a 30-to-45 minute motivational interviewing (MI) consultation; it is not a standalone tool.

Out-of-scope and prohibited uses

- • Use to deny, delay, or withdraw CGM access (the protocol explicitly states that the score does not affect allocation during the pilot phase).
- • Use in adults, in children younger than 10 years, or outside Oman, until external validation in those populations is satisfactory.
- • Use for any condition other than type 1 diabetes.
- • Use without an accompanying MI-trained clinician.
- • Use in unsupervised consumer settings (the model is not designed for direct-to-patient delivery).
- • Use of model outputs as the sole input to any automated insulin or device decision.

3. Training data summary

Source: pseudonymised records of children aged 10–18 years with type 1 diabetes who received a CGM through the Omani national rollout between July 2024 and February 2025, extracted from the Al Shifa national electronic health record (sub-study 1, n ≈ 1500), combined with structured face-to-face interview data collected in sub-study 2 (planned n = 240) and the dichotomous Optimizer / Sub-user outcome derived from device-recorded sensor wear at ≥ 3 months.

Candidate input features (pre-specified)

Domain	Variables (illustrative, finalised at lock)
Demographics	Age, sex, governorate of residence.
Clinical	Diabetes duration, baseline HbA1c, insulin modality (multiple daily injections vs pump), baseline weight, height, BMI z-score, blood pressure.
Laboratory	Lipid panel, renal function tests, liver function tests.
Family / context	Primary caregiver, family history of diabetes.
Psychosocial	BenCGM (perceived benefit of CGM), BurCGM (perceived burden of CGM), SEDM (self-efficacy), PAID-Peds (diabetes distress), RSQ-PD coping factors.
Behavioural	PAQ-A physical activity score, short dietary screener score.
Service	CGM device type, number of dispensations to date.

Outcome label: CGM Optimizer (≥ 6 days/week) vs CGM Sub-user (< 6 days/week or discontinued), measured from device records at ≥ 3 months from baseline, consistent with Messer et al (2022).

Geographic and demographic coverage (planned)

- • Coverage of all 11 Omani governorates, recruitment stratified proportional to CGM dispensations.
- • Age range 10–18 years (inclusive of both pre- and post-pubertal stages).
- • Balanced representation of sex anticipated based on the national T1DM register; final balance to be reported in the model card update at lock.
- • Insulin modality mix expected to reflect the national distribution at the time of the cohort (predominantly multiple daily injections, with a growing pump minority).

Data not included in training

- • No free-text qualitative content from sub-study 2 enters the predictive model; qualitative themes are used only to inform feature interpretation and MI content.
- • No continuous glucose readings, no insulin-pump telemetry, and no continuous sensor traces are used as model inputs (only the dichotomous adherence outcome derived from device records).
- • No genomic, social media, or geolocation data are used.

4. Model development methods (pre-specified)

- • Missing data: variables with > 40% missing excluded unless of strong prior clinical value; item-level missingness on validated scales handled with each scale's published rules; remaining missingness imputed by multiple imputation by chained equations (m = 20) under a missing-at-random assumption, with a tipping-point sensitivity analysis under missing-not-at-random.
- • Class imbalance: stratified resampling within cross-validation folds and class-weighted loss functions (inverse-frequency); SMOTE evaluated as a sensitivity analysis only.
- • Overfitting controls: nested cross-validation (outer 5-fold for performance estimation, inner 5-fold for hyperparameter tuning); L1/L2 regularisation for linear models; early stopping and shrinkage for boosted trees; dropout and weight decay for the neural network. Temporal partitioning of the training and test sets where feasible.
- • Feature selection: stability selection wrapper around L1-penalised logistic regression with 100 subsampled fits; features retained at ≥ 60% selection frequency, in addition to a clinically pre-specified core set.
- • Interpretability: final model deliberately sparse; SHAP values reported alongside predictions to support clinical interpretation.

5. Performance metrics (protocol stage)

The following metrics will be reported once the model is trained and locked. At present (protocol stage), no performance values are available.

Metric	Pre-specified threshold for acceptable performance
Area under the ROC curve (AUC)	≥ 0.70 on the internal hold-out partition.
Sensitivity (for Sub-user class)	≥ 0.70 at the selected operating point.
Specificity	≥ 0.60 at the selected operating point.
Brier score	Reported with 95% confidence interval; lower is better.
Calibration slope	Between 0.85 and 1.15 in the internal hold-out.
Calibration-in-the-large	$\lvert \text{intercept} \rvert \leq 0.10$ in the internal hold-out.
Decision-curve analysis	Net benefit > treat-all and treat-none across a clinically plausible threshold range (0.20–0.50).

Performance reporting plan by subgroup

All performance metrics above will additionally be reported separately for the following subgroups, with 95% confidence intervals:

Subgroup attribute	Strata
Sex	Female; Male.
Age band	10-13 years; 14-18 years.
Governorate	11 strata (Muscat, Dhofar, Musandam, Al Buraimi, Al Dakhiliyah, Al Batinah North, Al Batinah South, Al Sharqiyah North, Al Sharqiyah South, Al Dhahirah, Al Wusta).
Insulin modality	Multiple daily injections; pump.

A fairness audit will be triggered if the absolute difference in false-negative rate between any two strata of the same attribute exceeds 10 percentage points. If triggered, the model card will document the disparity, the mitigation applied (group-specific threshold adjustment or feature re-weighting), and the post-mitigation metrics.

6. Calibration plan

Calibration will be assessed before model lock using calibration plots, the Brier score, calibration-in-the-large, and calibration slope. If the raw model is mis-calibrated, isotonic regression or Platt scaling will be fitted on the inner cross-validation folds and re-evaluated on the outer fold. Decision-curve analysis will accompany the calibration report. Recalibration will be repeated at each external validation site before that site is permitted to use model outputs.

7. External validation plan

- • Internal validation: 20% held-out partition of the training data; nested cross-validation also reported.
- • Temporal validation: prospectively collected cohort enrolled at least three months after model lock, using identical data-collection forms and outcome definitions.
- • External multi-site replication: two Gulf neighbouring sites (institutions to be confirmed at the start of the pilot phase) using the same forms, the same outcome definition, and (where feasible) a federated analysis to avoid transfer of individual-level records.

Until external validation is satisfactory at one or more independent sites, OMNIdiasense outputs will be used as decision support only and will not influence CGM allocation.

8. Risk communication to families

- • The score is communicated to families as a categorical band (higher, moderate, lower), not as a raw probability.
- • Counselling makes explicit that the score is a research-stage estimate, not a diagnosis, and that it does not affect CGM access.
- • Families are told what the actionable behavioural recommendations are and that they may decline the recommendations without any consequence for routine care.
- • The communication template was reviewed by the parent advisory group convened in the first quarter of sub-study 2.

9. Data privacy and security

- • Identifiable data are stored only within the Ministry of Health Al Shifa environment behind the national firewall.
- • The analytic dataset shared with the AI team is pseudonymised; the re-identification key is held by the PI in a separate encrypted store.
- • Model training and inference take place on a Ministry of Health controlled environment; no patient data leave the national network for training or inference.
- • Access is role-based, with audit logging at the record level.
- • External validation uses federated analysis where technically feasible; otherwise a Data Transfer Agreement compliant with Royal Decree 6/2022 (Oman Personal Data Protection Law) and the host country's equivalent governs any transfer.

10. Known and anticipated limitations

- • The model is trained on Omani children aged 10–18 years; generalisability to younger children, adults, or other countries is unknown until external validation.
- • The Optimizer / Sub-user dichotomy at 3 months may not capture longer-term adherence trajectories.
- • Some psychosocial variables are self-reported and subject to social-desirability bias in a face-to-face interview setting.
- • Sample size of the pilot RCT (n = 50) limits the precision of effect-size estimates and may be underpowered for the planned subgroup-fairness audit in small strata.
- • Predictor distributions may drift over time as device generations change and clinical practice evolves; ongoing drift monitoring is required.
- • The model does not predict acute clinical events (DKA, severe hypoglycaemia) and must not be used as a safety-monitoring tool.

11. Maintenance and drift monitoring

- • Version control: every retraining produces a new model version; the deployed version is recorded with the prediction in the audit log.
- • Drift monitoring: population shift in input features and in outcome prevalence is reviewed by the Data Monitoring Committee every six months during the pilot.
- • Performance monitoring: AUC, calibration slope, calibration-in-the-large, and subgroup false-negative rate are re-estimated on a rolling 3-month window of post-deployment data.
- • Threshold review: the operating threshold is reviewed annually or whenever calibration drift exceeds the pre-specified tolerance, whichever is sooner.
- • Complaint handling: a clinician- and family-facing reporting channel is maintained for usability and concern reporting; complaints are reviewed at every DMC meeting.

12. Decommissioning conditions

The model will be decommissioned (i.e., withdrawn from clinical use and the recommendation engine deactivated) if any of the following are met. The decision is made by the Trial Steering Committee on the advice of the Data Monitoring Committee.

- • Persistent calibration drift outside the pre-specified tolerance (calibration slope outside 0.85–1.15 or calibration-in-the-large |intercept| > 0.10) over two consecutive monitoring cycles, not resolvable by recalibration.
- • Detection of a clinically meaningful subgroup disparity (false-negative-rate gap > 10 percentage points between any two strata) that cannot be reduced by mitigation within one monitoring cycle.
- • A device-related serious adverse event signal that is judged plausibly related to model-guided behavioural recommendations.
- • Failure of independent external validation at any planned external site, where failure is defined as AUC below 0.65, or a calibration slope outside the tolerance band, or a clinically meaningful subgroup disparity at that site.
- • Interruption of national CGM supply or material change in the eligibility pathway, such that the training distribution no longer reflects the deployment population.
- • Availability of a successor model (internal or external) that demonstrably outperforms OMNIdiasense on the pre-specified metrics in a head-to-head evaluation.
- • Withdrawal of ethics approval, or change in national data-protection law that the existing safeguards do not meet.

Decommissioning is reversible only after a documented retraining, recalibration, and ethics re-approval cycle. The decommissioning event itself is recorded in the Ministry of Health digital-health change log and reported in the next public update of this model card.

13. Quick-reference summary (model facts label)

Field	Value
Name and version	OMNIdiasense v0 (protocol stage).
Intended use	Pre-dispensing estimate of CGM Sub-user risk in Omani children aged 10-18 years with T1DM.

Users	Trained diabetes nurses and paediatric endocrinologists in MoH diabetes clinics.
Inputs	Demographics, clinical, laboratory, psychosocial (BenCGM, BurCGM, SEDM, PAID-Peds, RSQ-PD), behavioural (PAQ-A, dietary screener), service variables.
Output	Categorical risk band (higher, moderate, lower) and an MI-aligned recommendation set.
Use restriction	Decision support only. Does not affect CGM allocation. Not for use outside the studies described in the protocol until external validation.
Reporting standard	TRIPOD-AI (BMJ 2024).
Owner	Ministry of Health, Sultanate of Oman.
Date of this card	29 May 2026.

14. References for this model card

- Mitchell M, Wu S, Zaldivar A, Barnes P, Vasserman L, Hutchinson B, et al. Model cards for model reporting. Proceedings of the Conference on Fairness, Accountability, and Transparency, 2019:220–229. doi:10.1145/3287560.3287596
- Sendak MP, Gao M, Brajer N, Balu S. Presenting machine learning model information to clinical end users with model facts labels. NPJ Digit Med. 2020;3:41. doi:10.1038/s41746-020-0253-3
- Collins GS, Moons KGM, Dhiman P, Riley RD, Beam AL, Van Calster B, et al. TRIPOD+AI statement: updated guidance for reporting clinical prediction models that use regression or machine learning methods. BMJ. 2024;385:e078378. doi:10.1136/bmj-2023-078378
- Messer LH, Cook PF, Lowe NK, Hood KK, Driscoll KA, Hernandez TL. Predicting optimal use of continuous glucose monitors in adolescents with type 1 diabetes: it's about benefit and burden. J Pediatr Nurs. 2022;62:23–29. doi:10.1016/j.pedn.2021.11.016
- Royal Decree No. 6/2022 promulgating the Personal Data Protection Law of the Sultanate of Oman. Official Gazette, 9 February 2022.
