# Supplementary material for: An AI-Assisted Tool to Predict Continuous Glucose Monitor Adherence in Children With Type 1 Diabetes in Oman: Protocol for a Multiphase Mixed Methods Translational Study
Source: JMIR Res Protoc. 2026 Jul 13;15:e99626. doi: 10.2196/99626 (PMC13408470; doi:10.2196/99626)
Supplement: Multimedia Appendix 8 [file resprot_v15i1e99626_app8.pdf]

Checklist 4. Brief Motivational Interviewing Consultation Guide and Fidelity Rubric

Protocol: *An Artificial Intelligence-Assisted Tool to Predict Continuous Glucose Monitor Adherence in Children with Type 1 Diabetes in Oman: Protocol for a Multi-Phase Mixed Methods Translational Study* (ISRCTN15827616).

Purpose. This checklist standardises the three motivational interviewing (MI) consultations delivered at baseline, 3 months, and 6 months in sub-study 3, and provides the fidelity rubric used for the 10% per-interventionist per-quarter audio-recorded session sampling described in the Methods. It is anchored to Miller and Rollnick's four MI processes (Engaging, Focusing, Evoking, Planning) and to the Motivational Interviewing Treatment Integrity manual, version 4.2.1 (MITI 4.2.1).

Intended user. Diabetes specialist nurses, paediatric endocrinologists, and study Research Managers who have completed the project's three-day training programme. The guide is designed for a 30-45-minute consultation in Arabic or English, in dyadic (adolescent + caregiver) or paired-then-separate format.

Part A. Operational Principles

1. **Spirit of MI.** Every consultation must reflect the four foundational elements: *partnership, acceptance, compassion,* and *evocation.* The clinician is a guide, not a director. The family's autonomy, expertise about their own life, and right to refuse any element of the recommendation are honoured throughout.
2. **OARS skills** are the building blocks of every section: **O**pen questions, **A**ffirmations, **R**eflections (simple and complex), **S**ummaries.
3. **Change talk first; sustain talk normalised.** Ambivalence is expected and welcomed. The clinician selectively reinforces *change talk* (Desire, Ability, Reasons, Need, Commitment, Activation, Taking steps, DARN-CAT) and softens *sustain talk* without confronting it.
4. **Elicit-Provide-Elicit.** When sharing the OMNIdiasense risk score or any clinical advice, ask permission first, share clearly, and ask what the family makes of it.
5. **Cultural and linguistic responsiveness.** Use the family's preferred language; respect Omani family hierarchy by addressing parents as well as the adolescent; offer same-sex interviewers where requested; frame recommendations around family, school, and community contexts (Eid, fasting, weddings, sleepovers); avoid jargon and acronyms.
6. **Documentation.** Every consultation is logged in the session log and entered within the routine health information system.

Part B. Session Structure

The same six-step architecture is applied at each visit. Items in **bold** are mandatory and contribute to the fidelity rubric in Part C.

B1. Baseline session (~45 min)

Step	MI process	Time	What to do
1. Welcome and orientation	Engaging	3 min	**Greet the family by name; introduce yourself and your role; state the purpose and length of the conversation; ask permission to proceed and to audio-record.**
2. Build rapport	Engaging	5-7 min	Open question on the family's day-to-day life with diabetes. **Provide ≥2 simple or complex reflections.** Do not lead. Affirm at least one strength (e.g., "I can see how much thought you have already put into this").
3. Agenda setting	Focusing	3-5 min	Ask the family what feels most important right now (CGM wear, school, sleep, food, sport, family stress). Use a bubble sheet if helpful. **Negotiate one to two focal topics; record them in the session log.**
4. Sharing the OMNIdiasense risk score	Evoking	8-10 min	Use Elicit-Provide-Elicit. *Elicit:* "What do you already know about how the CGM has been going?" *Provide* (with permission): score, the two strongest contributing factors, and what they mean in plain language. *Elicit:* "What do you make of that?" **Avoid persuasion or warning.**
5. Building motivation and confidence	Evoking	10-12 min	Use the Importance and Confidence rulers (0-10 each): "On a scale of 0 to 10, how important is it for you that the CGM is worn most days? Why not lower? What would help move it higher?" Reflect change talk; affirm strengths; explore the imagery of life with and without the CGM.
6. Plan and close	Planning	5-8 min	If readiness is present, **co-develop one specific, achievable adherence step for the next 4-6 weeks** (when, where, who, how). Ask "What might get in the way?" and plan around it. **Summarise back; confirm next contact; thank the family.**

B2. 3-month session (~30 min)

Compressed version of the baseline structure. Mandatory steps:

1. **Re-engage** with an open question and at least one reflection.
2. **Review** the previous adherence step (what worked, what did not, what was learned). Affirm effort regardless of outcome.
3. **Re-share the updated OMNIdiasense score** using Elicit-Provide-Elicit.
4. **Re-evoke** with a single ruler (Importance OR Confidence, whichever was lower at baseline).

5. **Refine the plan** (keep, adjust, or replace the previous step).
6. **Summarise and close.**

B3. 6-month session (~30 min)

Same structure as the 3-month session, with two additions:

7. **Generalisation question:** "What from this work do you want to keep, even after the project ends?"
8. **Sustainability plan:** identify one family routine and one clinic-side routine that will support continued CGM use.

Part C. Brief MI Fidelity Rubric

This rubric is applied to a randomly sampled random audio recording from each interventionist, each quarter. Each rubric is completed independently by two trained coders; discrepancies are resolved by a third senior coder. A coding window of 20 contiguous minutes from the middle of the session is used unless the session is shorter, in which case the entire session is coded.

C1. Global ratings (5-point scale)

For each global dimension, assign a single integer score for the coded segment.

Dimension	1-Low	2	3-Acceptable	4	5-High
**Cultivating Change Talk (CC)** *Encourages the family's own language in favour of CGM wear and confidence for making that change.*	Misses or discourages change talk	Occasional, mostly missed opportunities	Some attention; modest evocation of change talk	Consistently invites and reinforces change talk	Skilfully evokes and develops change talk; pace and depth are excellent
**Softening Sustain Talk (SS)** *Avoids a focus on the reasons against wearing the CGM or on maintaining the status quo.*	Reinforces sustain talk; argues against it	Frequent over-attention to sustain talk	Some balance; mixed responses	Generally, avoids dwelling on sustain talk; redirects skilfully	Smoothly de-emphasises sustain talk without confrontation
**Partnership (P)** *Conveys understanding that expertise about change resides mostly with the family.*	Acts as the expert; one-up stance	Token collaboration	Mixed; some power-sharing	Clear collaborative stance	Family is the active partner throughout

**Empathy (E)** *Understands or makes an effort to grasp the family's perspective and experience.*	Little interest in the family's view	Sporadic understandin g	Active attempts to understand	Clearly grasps multiple layers	Deep, accurate understandin g consistently demonstrated

The **Relational global** = (Partnership + Empathy) / 2; the **Technical global** = (Cultivating + Softening) / 2.

C2. Study-specific items

Each item is scored 0 = not done, 1 = partially done, 2 = clearly done. A session must score ≥ 1 on **every** item.

#	Study-specific item	0	1	2
S1	Audio-recording consent obtained verbally and confirmed at start of recording	☐	☐	☐
S2	Adolescent and caregiver were each given an opportunity to speak	☐	☐	☐
S3	Agenda was negotiated and at least one focal topic recorded in the session log	☐	☐	☐
S4	OMNIdiasense risk score was shared using Elicit-Provide-Elicit, with explicit permission	☐	☐	☐
S5	At least one ruler (Importance or Confidence) was used	☐	☐	☐
S6	A specific, time-bound adherence step was co-developed (or, if not ready, this was named explicitly without coercion)	☐	☐	☐
S7	Cultural and linguistic responsiveness observed (preferred language, no jargon, family context honoured)	☐	☐	☐
S8	Closing summary was delivered and next contact confirmed	☐	☐	☐

**Overall fidelity outcome** *(Below threshold / Beginning proficiency / Competent)*	
Coder comments and recommended supervision focus	

C3. Quarterly supervisor feedback

Each interventionist receives a one-page individualised feedback report and a 30-minute supervision call.

Part D. Reporting and record-keeping

- **Session log fields:** session ID, date, duration, attendees, agenda topics, ruler scores, agreed step, next contact, recording yes/no, consent confirmed. All outputs will be stored in encrypted files with the independent quality officer for 24 months and then destroyed, per the data-management plan.

Selected references

1. Miller WR, Rollnick S. *Motivational Interviewing: Helping People Change*. 3rd ed. New York, NY: Guilford Press; 2013.
2. Moyers TB, Rowell LN, Manuel JK, Ernst D, Houck JM. The Motivational Interviewing Treatment Integrity Code (MITI 4): rationale, preliminary reliability and validity. *J Subst Abuse Treat*. 2016;65:36-42. doi:10.1016/j.jsat.2016.01.001
3. Moyers TB, Manuel JK, Ernst D. *Motivational Interviewing Treatment Integrity Coding Manual 4.2.1*. Albuquerque, NM: Center on Alcoholism, Substance Abuse, and Addictions, University of New Mexico; 2014. URL: https://casaa.unm.edu/assets/docs/miti4_21.pdf
4. Powell PW, Hilliard ME, Anderson BJ. Motivational interviewing to promote adherence behaviors in pediatric type 1 diabetes. *Curr Diab Rep*. 2014;14(10):531. doi:10.1007/s11892-014-0531-z
5. Channon SJ, Huws-Thomas MV, Rollnick S, Hood K, Cannings-John RL, Rogers C, Gregory JW. A multicenter randomized controlled trial of motivational interviewing in teenagers with diabetes. *Diabetes Care*. 2007;30(6):1390-1395. doi:10.2337/dc06-2260
6. Naar S, Suarez M. *Motivational Interviewing with Adolescents and Young Adults*. New York, NY: Guilford Press; 2021.
