# Supplementary material for: An AI-Assisted Tool to Predict Continuous Glucose Monitor Adherence in Children With Type 1 Diabetes in Oman: Protocol for a Multiphase Mixed Methods Translational Study
Source: JMIR Res Protoc. 2026 Jul 13;15:e99626. doi: 10.2196/99626 (PMC13408470; doi:10.2196/99626)
Supplement: Multimedia Appendix 9 [file resprot_v15i1e99626_app9.docx]

Satisfaction Exit Surveys (Multimedia Appendix 6)

Two structured questionnaires share a common core architecture, allowing item-level comparison between **parents/caregivers** and **healthcare workers**. Both versions are administered at the **6-month** and **12-month** follow-up time points. Likert items use a balanced 5-point scale (Strongly disagree (1), Disagree (2), Neutral (3), Agree (4), Strongly agree (5)) with a “Not applicable / I don’t know” option. Free-text items are optional but encouraged.

5.1 Parent/Caregiver Satisfaction Survey

Section A — Demographic and contextual information

- A1. Age of the child (years): ___
- A2. Sex of the child: ☐ Male ☐ Female
- A3. Governorate: ___
- A4. Duration of type 1 diabetes (years): ___
- A5. Insulin modality: ☐ Multiple daily injections ☐ Insulin pump
- A6. Months since first CGM dispensed: ___
- A7. Relationship to the child: ☐ Mother ☐ Father ☐ Other guardian (specify)
- A8. Highest education level of respondent: ☐ Primary ☐ Secondary ☐ Diploma/Bachelor ☐ Postgraduate
- A9. Preferred language for healthcare information: ☐ Arabic ☐ English ☐ Both

Section B Usability of OMNIdiasense

B1. The OMNIdiasense questions were easy to understand. B2. The time taken to complete the tool was reasonable. B3. The recommendations from the tool were clear to me. B4. The Arabic-language version was natural and culturally appropriate.

Section C Trust and credibility

C1. I trust the predictions made by the OMNIdiasense tool. C2. The recommendations matched my own sense of how my child uses the CGM. C3. The clinical team explained the tool’s predictions in a way that respected my role as a parent.

Section D Perceived benefit

D1. The tool helped me understand my child’s CGM use better. D2. The motivational interviewing sessions helped my family adjust how we use the CGM. D3. Since starting the project, my child wears the sensor more consistently. D4. Since starting the project, our family stress around diabetes has decreased. D5. Since starting the project, communication with the diabetes clinic has improved.

Section E Safety, privacy, and ethics

E1. I felt that my child’s information was kept private and secure. E2. I felt able to ask questions or refuse any part of the project at any time. E3. The consent process was clear and complete.

Section F Equity and access

F1. The tool worked well in our local language and cultural context. F2. Distance to the diabetes clinic was a barrier to using the tool. *(reverse-scored)* F3. The cost of CGM supplies has been a barrier for our family. *(reverse-scored)*

Section G Open-ended

G1. What did you like most about the OMNIdiasense tool and the motivational interviewing sessions? G2. What did you find most difficult or unhelpful? G3. What would you change to make the tool work better for Omani families? G4. Would you recommend this approach to other families with a child with type 1 diabetes? Why or why not?

Section H Overall satisfaction (single-item global rating)

H1. Overall, how satisfied are you with the OMNIdiasense project? *0 (not at all) — 10 (extremely)*

Section I Net Promoter-style item

I1. How likely are you to recommend this approach to another family with type 1 diabetes? *0–10*

5.2 Healthcare Worker Satisfaction Survey

Section A Demographic and professional information

- A1. Age (years): ___
- A2. Sex: ☐ Male ☐ Female ☐ Prefer not to say
- A3. Profession: ☐ Diabetes specialist nurse ☐ Pediatric endocrinologist ☐ General pediatrician ☐ Pharmacist ☐ Other (specify)
- A4. Years of experience caring for children with T1DM: ___
- A5. Governorate of practice: ___
- A6. Number of OMNIdiasense assessments personally completed: ___
- A7. Hours of MI training completed: ___

Section B Usability and integration

B1. The OMNIdiasense interface is intuitive to navigate. B2. The tool integrates well with my existing clinic workflow. B3. The time required per patient is acceptable. B4. Documentation requirements are reasonable.

Section C Clinical utility and trust

C1. The tool’s adherence-risk predictions match my clinical judgement. C2. The personalised recommendations are clinically appropriate. C3. The tool helps me identify families who need extra support. C4. The tool supports — rather than replaces — clinical decision-making.

Section D Perceived benefit on care

D1. The tool has improved how I prepare patients for CGM dispensing. D2. The motivational interviewing component has strengthened my consultations. D3. Patients seem more engaged when the tool is used. D4. Adherence among my patients has improved since the project started.

Section E Safety, ethics, and equity

E1. The tool handles patient data securely and confidentially. E2. The tool performs equally well across age, sex, and socioeconomic groups in my catchment. E3. The Arabic-language version is clinically and culturally accurate. E4. I have had at least one situation where my clinical judgement overrode the tool’s recommendation. (Yes / No — if yes, please describe in G2.)

Section F Training and support

F1. The training I received prepared me to use the tool confidently. F2. Technical support is responsive when I need it. F3. There is sufficient peer learning and supervision available.

Section G Open-ended

G1. What works best about the OMNIdiasense tool in your daily practice? G2. Describe a situation in which you disagreed with or modified the tool’s recommendation. G3. What changes to the tool, the training, or the workflow would have the biggest impact? G4. What would be needed for sustained use of OMNIdiasense after the project ends?

Section H Overall satisfaction

H1. Overall satisfaction with OMNIdiasense in your clinical practice: *0 (not at all) — 10 (extremely)*

Section I Adoption intent

I1. How likely are you to continue using this tool if available after the project? *0–10* I2. How likely are you to recommend this approach to colleagues in another clinic? *0–10*

Scoring and interpretation guidance

- For each domain (B, C, D, E, F), compute the mean Likert score; interpret ≥4.0 as favourable.
- Reverse-scored items (e.g., F2 in 5.1, items prefixed *(reverse-scored)*) are recoded prior to mean calculation.
- The single-item global rating (H1) and the 0–10 recommendation item (I1, I2) provide a parsimonious overall metric and a Net Promoter-style score, respectively.
- Free-text responses (G items) are analysed using rapid framework analysis aligned with the qualitative topic guide in §4.
