# Supplementary material for: An AI-Assisted Tool to Predict Continuous Glucose Monitor Adherence in Children With Type 1 Diabetes in Oman: Protocol for a Multiphase Mixed Methods Translational Study
Source: JMIR Res Protoc. 2026 Jul 13;15:e99626. doi: 10.2196/99626 (PMC13408470; doi:10.2196/99626)
Supplement: Multimedia Appendix 10 [file resprot_v15i1e99626_app10.pdf]

Critical Appraisal & Supplementary Materials

Protocol appraised: *An Artificial Intelligence-Assisted Tool to Predict Continuous Glucose Monitor Adherence in Children with Type 1 Diabetes in Oman: Protocol for a Multi-Phase Mixed Methods Translational Study* (JMIR Research Protocols submission, April 2026; ISRCTN15827616).

1. SPIRIT 2025 Checklist 1

The SPIRIT 2025 statement (Chan et al., *BMJ* 2025;389:e081477) replaces SPIRIT 2013. It contains 34 items in 9 domains and is the current standard for protocols of randomised trials. Although the OMNIdiasense project is a multi-phase translational programme rather than a stand-alone RCT, the embedded pilot RCT (sub-study 3) requires SPIRIT compliance. The retrospective cohort (sub-study 1) and cross-sectional mixed methods study (sub-study 2) are appraised separately under STROBE and COREQ below.

#	SPIRIT 2025 item	Description	Reported?	Page(s)
**Administrative information**				
1	Title	Descriptive title with study design and population	Yes	p. 1
2a	Trial registration	Trial registry, registration number, date	Yes (ISRCTN15827616)	p. 2 (abstract); p. 11 (Results)
2b	Trial registration data set	WHO Trial Registration Data Set or equivalent	Partial — registry ID provided; full dataset items not reproduced in the manuscript	p. 2
3	Protocol version	Date and version identifier	Not reported	—
4	Funding	Sources and types of financial,	Yes (Ministry of Higher Education, Research and Innovation;	p. 11 (Results); p. 12 (Funding)

		material, and other support	Agreement RIA/SRP/MoH/25/01)	
5a	Roles and responsibilities — contributorship	Names, affiliations, and roles of contributors	Yes	p. 1 (authors); p. 12 (Authors' Contributions)
5b	Sponsor and funder roles	Sponsor/funder role in design, conduct, analysis, reporting; statement of independence	Partial — funder role declared as none in Acknowledgments, but a formal sponsor section is missing	p. 12
5c	Committees	Composition, roles, responsibilities of coordinating centre, steering committee, endpoint adjudication committee, data management team, and other individuals overseeing the trial	Partial — Research Manager network described; no Trial Steering Committee, Endpoint Adjudication Committee, or Data Monitoring Committee specified	p. 5
**Introduction**				
6a	Background and rationale	Description of research question and justification for undertaking the trial; reference to relevant studies and any systematic review demonstrating equipoise	Yes	p. 3-5

6b	Choice of comparators	Explanation for choice of comparators	Yes (usual care + standard CGM dispensing pathway)	p. 8-9
7	Objectives	Specific objectives or hypotheses	Yes	p. 4-5
8	Trial design	Description of trial design (e.g., parallel group, crossover, factorial, single group), allocation ratio, framework (e.g., superiority, equivalence, non-inferiority)	Yes (parallel-group, 1:1 allocation, pilot/feasibility framework)	p. 8-9
**Methods: Participants, interventions and outcomes**				
9	Study setting	Description of study settings and list of countries where data will be collected	Yes (11 governorates of Oman; Al Shifa HIS)	p. 5
10	Eligibility criteria	Inclusion and exclusion criteria; criteria for individuals performing interventions	Yes for participants; criteria for interventionists (training described)	p. 6, p. 9
11a	Interventions: description	Sufficient detail to allow replication, including how and when administered	Yes (OMNIdiasense + 3 motivational interviewing sessions at	p. 9

			baseline, 3, 6 months)	
11b	Interventions: modifications	Criteria for discontinuing or modifying interventions for a given trial participant	Not reported	—
11c	Interventions: adherence	Strategies to improve adherence to intervention protocols and procedures for monitoring adherence	Partial — MI consultations support adherence but no fidelity-monitoring procedure is described	p. 9
11d	Interventions: concomitant care	Relevant concomitant care and interventions that are permitted or prohibited during the trial	Not reported	—
12	Outcomes	Primary, secondary, and other outcomes including the specific measurement variable, analysis metric, method of aggregation, and time point of interest	Yes	p. 9-10; Table 1 p. 18
13	Participant timeline	Schedule of enrolment, interventions, assessments, and visits — figure recommended	Yes (Table 1 schedule of assessments; SPIRIT-style figure not provided)	p. 18

14	Sample size	Estimated number of participants needed to achieve study objectives, with calculation and rationale	Yes (240 for sub-study 2; 100 single-arm + 50 RCT for sub-study 3)	p. 7, p. 9
15	Recruitment	Strategies for achieving adequate participant enrolment	Yes (RM-led, formal invitation, governorate stratification)	p. 9
**Methods: Assignment of interventions**				
16a	Allocation: sequence generation	Method of generating allocation sequence	Yes (computer-generated 1:1 by independent statistician)	p. 10
16b	Allocation concealment mechanism	Method to conceal allocation sequence	Yes (sequentially numbered opaque sealed envelopes)	p. 10
16c	Allocation: implementation	Who will generate, enrol, and assign	Partial — independent statistician generates; person enrolling is implied (RMs) but not explicit	p. 10
17a	Blinding (masking): who	Who will be blinded after assignment	Yes (outcome assessors and analyst blinded; participants/interventionists not blinded)	p. 10
17b	Blinding: emergency unblinding	Procedure to reveal allocation during the trial	Not applicable (open-label intervention; no	—

			pharmacological masking)	
**Methods: Data collection, management, and analysis**				
18a	Data collection methods	Plans for assessment and collection of outcome, baseline, and other trial data	Yes	p. 5-9; p. 18
18b	Data collection: retention	Plans to promote participant retention and complete follow-up	Partial — MI sessions inherently support retention; no explicit retention strategy	p. 9
19	Data management	Plans for data entry, coding, security, storage, and archiving	Partial — encrypted MoH systems and de-identified analytic datasets noted	p. 10
20a	Statistical methods: outcomes	Statistical methods for analysing primary and secondary outcomes	Yes	p. 6, p. 7, p. 10
20b	Statistical methods: subgroup analyses	Methods for any additional analyses (subgroup, adjusted)	Not reported	—
20c	Statistical methods: missing data	Definition of analysis population and methods to handle missing data and	Yes (multiple imputation under MAR, sensitivity analyses, ITT)	p. 10

		protocol non-adherence		
**Methods: Monitoring**				
21a	Data monitoring committee (DMC)	Composition, role, and reporting structure of the DMC; statement on independence; reference to charter	Not reported	—
21b	Interim analyses and stopping rules	Description of interim analyses and stopping guidelines	Not reported	—
22	Harms	Plans for collecting, assessing, reporting, and managing solicited and spontaneously reported adverse events and other unintended effects	Not reported	—
23	Auditing	Frequency and procedures for auditing trial conduct, if any, and whether the process will be independent	Not reported	—
**Ethics and dissemination**				
24	Research ethics approval	Plans for seeking research ethics	Yes (MoH/CSR/24/29506)	p. 2; p. 10

		committee/IRB approval		
25	Protocol amendments	Plans for communicating important protocol modifications to relevant parties (e.g., investigators, REC/IRB, trial participants, trial registries, journals, regulators)	Not reported	—
26a	Consent or assent	Procedures for obtaining informed consent from potential trial participants or authorised surrogates	Yes (parental written consent, child assent, Multimedia Appendix 3)	p. 9; p. 10
26b	Consent or assent: ancillary studies	Additional consent provisions for ancillary studies, if applicable	Not applicable (no ancillary studies planned)	—
27	Confidentiality	How personal information will be collected, shared, and maintained to protect confidentiality before, during, and after the trial	Yes (encrypted MoH systems, de-identified datasets, restricted reidentification keys)	p. 10
28	Declaration of interests	Financial and other competing interests for	Yes (declared none)	p. 13

		principal investigators for the overall trial and each study site		
29	Access to data	Statement of who will have access to the final trial dataset, and disclosure of contractual agreements that limit such access	Partial — Data Availability statement provided; site-level access not specified	p. 12
30	Ancillary and post-trial care	Provisions for ancillary and post-trial care, and for compensation to those who suffer harm from trial participation	Not reported	—
31a	Dissemination policy: trial results	Plans for investigators and sponsor to communicate trial results to participants, healthcare professionals, the public, and other relevant groups (e.g., via publication, results database, lay summaries)	Partial — publication timeline mentioned; lay/participant dissemination plan absent	p. 11
31b	Dissemination policy: authorship	Authorship eligibility guidelines and any intended use of	Partial — authorship covered; professional	p. 12

		professional writers	writer use not addressed	
31c	Dissemination policy: reproducible research	Plans, if any, to grant public access to the full protocol, participant-level dataset, and statistical code	Partial — Data Availability statement; protocol publication planned; statistical code not addressed	p. 12
**Appendices**				
32	Informed consent materials	Model consent form and other related documentation given to participants and authorised surrogates	Yes (Multimedia Appendix 3)	p. 13
33	Biological specimens	Plans for collection, laboratory evaluation, and storage of biological specimens for genetic or molecular analysis in the current trial and for future use, if applicable	Not applicable (no biological specimen banking)	—
34	Patient and public involvement	Plans for involvement of patients/public in trial design, conduct, analysis, reporting, and dissemination	Yes	p. 10

SPIRIT 2025 summary. Of the 34 items (with sub-items, 53 reportable elements), the protocol fully reports 22, partially reports 11, and does not report 12. The most important

gaps to address before submission are items **3, 5b, 5c, 11b, 11d, 21a, 21b, 22, 23, 25, 30**. These are addressed in §6 below (“Recommended additions to the protocol”).
