# Supplementary material for: An AI-Assisted Tool to Predict Continuous Glucose Monitor Adherence in Children With Type 1 Diabetes in Oman: Protocol for a Multiphase Mixed Methods Translational Study
Source: JMIR Res Protoc. 2026 Jul 13;15:e99626. doi: 10.2196/99626 (PMC13408470; doi:10.2196/99626)
Supplement: Multimedia Appendix 11 [file resprot_v15i1e99626_app11.pdf]

Sultanate of Oman
Ministry of Health
Directorate General of Planning & Studies
Centre of Studies and Research

سلطنة عمان
وزارة الصحة
المديرية العامة للتخطيط والدراسات
مركز الدراسات والبحوث

Ref.: MoH/DGPS/CSR/PROPOSAL_ APPROVED/18/2025

Date: 12/02/2025

الرقم : ....

التاريخ : ....

الموافق : ....

Dr Thamra Al Ghafri
Principal Investigator

<u>Study Title</u>: Utilizing an AI-assisted tool to Predict the Behaviour of Children with Type 1 Diabetes for Optimal Use of Sensor Technology in Oman: A Multi-phase Translational Research Project.

<u>Proposal ID</u>: MoH/CSR/24/29506

After compliments,

We are pleased to inform you that your research proposal '**Utilizing an AI-assisted tool to Predict the Behaviour of Children with Type 1 Diabetes for Optimal Use of Sensor Technology in Oman: A Multi-phase Translational Research Project**' has been approved by the Health Studies and Research Approval Committee (HSRAC), Ministry of Health.

The HSRAC should be notified in case of any changes or significant deviation from the approved proposal, otherwise this approval will be deemed invalid. On completion of the study, you are required to provide a copy of the final report within 2 months to the Centre of Studies and Research in Ministry of Health.

Regards,

Dr Issa Al-Salmi
Acting Chairperson
Health Studies and Research Approval Committee (HSRAC)
Ministry of Health, Sultanate of Oman

Cc: Day file

www.mohcsr.gov.om : الموقع الإلكتروني

P.O. Box: 393, Postal code 100, Muscat, Tel:22357280, Fax:22357296

ص . ب : ٣٩٣ ، الرمز البريدي : ١٠٠، مسقط ، هاتف : ٢٢٣٥٧٢٨٠، فاكس : ٢٢٣٥٧٢٩٦
