# Supplementary material for: An AI-Assisted Tool to Predict Continuous Glucose Monitor Adherence in Children With Type 1 Diabetes in Oman: Protocol for a Multiphase Mixed Methods Translational Study
Source: JMIR Res Protoc. 2026 Jul 13;15:e99626. doi: 10.2196/99626 (PMC13408470; doi:10.2196/99626)
Supplement: Multimedia Appendix 12 [file resprot_v15i1e99626_app12.pdf]

Sultanate of Oman
Ministry of Health
Directorate General of Planning & Studies
Centre of Studies and Research

سلطنة عمان
وزارة الصحة
المديرية العامة للتخطيط والدراسات
مركز الدراسات والبحوث

Ref.: .... الرقم : ....

Date: 30 December 2025 التاريخ : ....

الموافق : ....

Dr Saud Al-Harthi
Principal Investigator

Subject: Strategic Research Programme for Health 2025 Grant Award Letter

After greetings,

Congratulations! Your proposal entitled "**Utilizing an AI-assisted tool to Predict the Behavior of Children with Type 1 Diabetes for Optimal Use of Sensor Technology in Oman: A Multi-phase Translational Research Project**" submitted to the 'Artificial Intelligence (AI) in healthcare supporting decision-making' research theme of the Strategic Research Programme (SRP) for Health 2025 Cycle by the Ministry of Higher Education, Research and Innovation (MoHERI), has been approved for funding.

The research agreement sub-contract will be sent to your institution which will state the guidelines you are obliged to comply with as set out by the MoHERI and Ministry of Health.

We, at the National Health Research Centre, along with the Strategic Research Programme Committee, assure to support you with your efforts during the course of your research grant. We also take this opportunity to wish you and your research team a fruitful and successful research project, and look forward to the final report at the end of the project.

Yours sincerely,

CENTER OF STUDIES & RESEARCH
DIRECTORATE GENERAL OF PLANNING AND STUDIES
SULTANATE OF OMAN - MINISTRY OF HEALTH

Dr Zuhair Al-Sulti
Focal Person, Strategic Research Programme for Health
National Health Research Centre, Ministry of Health
